# ReLeaf-SAM: reliability-guided detail compensation for SAM-based plant disease segmentation

**DOI:** 10.3389/fpls.2026.1865649

**Published:** 2026-07-08

**Authors:** Fuyong Liu, Yaxin Hu, Qian Zhou, Hua Zou

**Affiliations:** 1 School of Information Science and Engineering, Xinjiang College of Science and Technology, Korla, Xinjiang, China; 2 School of Computer Science, Wuhan University, Wuhan, Hubei, China

**Keywords:** detail compensation, plant disease segmentation, precision agriculture, segment anything model, uncertainty-aware fusion

## Abstract

Accurate lesion segmentation is essential for automated plant disease analysis in precision agriculture. Although the Segment Anything Model (SAM) exhibits strong generalization ability, its direct application to plant disease images in natural field environments remains challenging due to cluttered backgrounds, dense leaf veins, uneven illumination, and frequent occlusions. In particular, SAM mainly relies on global structural cues and is often insufficiently sensitive to subtle lesion textures and weak local details, which can result in missed small or early-stage lesions and inaccurate boundary delineation. To address these limitations, we enhance SAM with a disease-specific detail compensation module for plant disease lesion segmentation. A ResNet-50based branch is employed to extract fine-grained local texture features that are difficult for SAM to capture. These fine-grained features are fused with SAM encoder representations and then injected into the SAM decoder, enabling more accurate lesion prediction while preserving SAM’s strong global modeling capability. More importantly, we propose a reliability-guided variational fusion framework to further improve the interaction between heterogeneous features. Specifically, instead of conventional similarity or addition-based fusion, we introduce an uncertainty-aware variational fusion strategy that explicitly quantifies the confidence of each feature stream. An uncertainty encoder models feature distributions probabilistically, and a variational fusion module dynamically assigns higher weights to more reliable features while suppressing uncertain or interfering responses. In addition, Kullback-Leibler divergence regularization is introduced to stabilize cross-feature alignment and improve fusion robustness. Extensive experiments on PlantSeg, PlantDoc-Seg, and ATLDSD demonstrate that the proposed method outperforms state-of-theart approaches, achieving DSC scores of 81.05%, 91.12%, and 88.27%, respectively. The proposed method addresses SAM’s weakness in fine-grained disease feature extraction, accurately identifies early and small lesions, and delivers reliable segmentation for field plant disease automatic diagnosis.

## 1 Introduction

Plant diseases substantially reduce crop productivity and remain a major challenge to sustainable agriculture (Bischoff et al., 2021; Lu et al., 2023). Accurate lesion segmentation is a key step in automated plant disease analysis, as it enables precise characterization of lesion morphology, size, and spatial distribution for subsequent diagnosis and disease management (Lu et al., 2024; Das et al., 2025). However, manual annotation of disease lesions is time-consuming, labor-intensive, and often affected by subjective bias, which limits its practicality in large-scale agricultural applications (Singh and Misra, 2017; Liu et al., 2024). In contrast, computer vision-based segmentation methods provide a more efficient and consistent solution for lesion analysis while reducing reliance on expert annotation (Revathi and Hemalatha, 2012; Zhang et al., 2023; Lu et al., 2025).

Recent studies have explored deep learning-based methods for plant disease lesion segmentation and achieved promising results on benchmark datasets.

**Figure 1:**
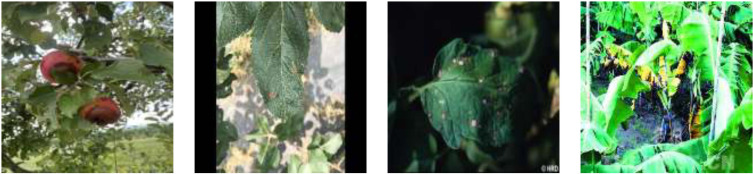
Examples of leaves with cluttered backgrounds, dense leaf veins, uneven illumination, and occlusions.

Several works have extended DeepLabv3/DeepLabv3+ architectures specifically for plant disease segmentation. Pal et al (Pal et al., 2025). combines MAML with DeepLabV3 for few-shot segmentation and disease severity assessment. Sharma et al (Sharma et al., 2025). proposes DBA-DeepLab, which integrates dual backbones of ResNet-50 and EfficientNet-B3 with CBAM attention. These studies underscore the ongoing value of CNN-based segmentation models in plant pathology. AISOA-SSformer (Dai et al., 2024) achieves accurate lesion segmentation by utilizing an improved Transformer architecture for leaf disease segmentation. DTM-Unet (Shi et al., 2026) fuses DenseNet and Transformer to achieve accurate identification and quantitative segmentation of cucumber leaf diseases. Besides, some semi-supervised methods, such as SSGAN (Zhao et al., 2025a), has emerged as a pivotal strategy to alleviate the high cost of annotation, and introduced Boundary Feature Attention Module to provide valuable local texture representation for accurate lesion localization. However, their performance often deteriorates in real field environments, where cluttered backgrounds, dense leaf veins, uneven illumination, and occlusions make lesion regions difficult to distinguish. **Figure 1** shows some examples of such challenging cases.

**Figure 2:**
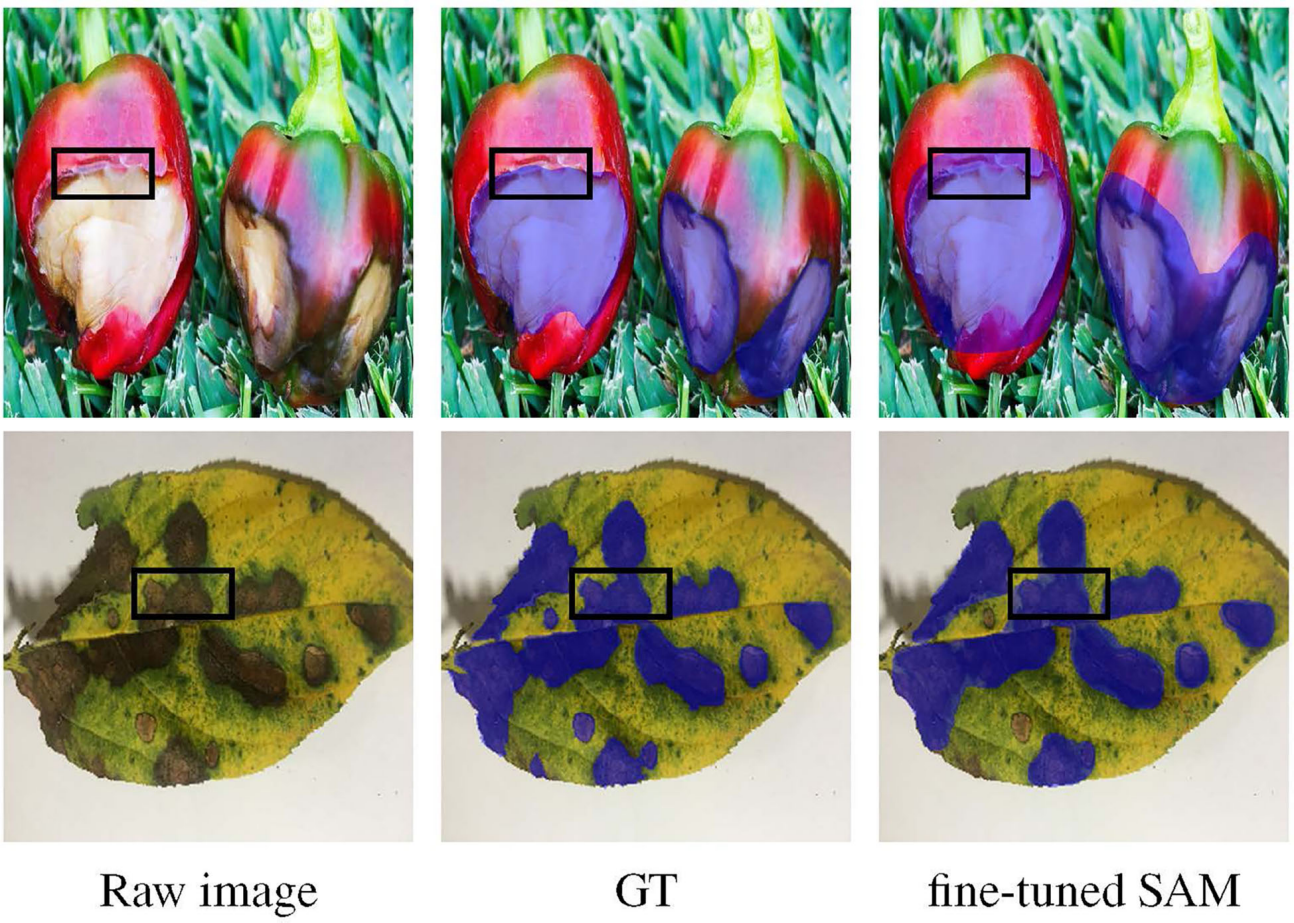
Results of fine-tuned SAM on leaf disease segmentation. GT means ground truth.

These limitations motivate the exploration of foundation models with stronger generalization ability. In particular, the Segment Anything Model (SAM) (Kirillov et al., 2023) has shown impressive performance in generic segmentation tasks due to large-scale pre-training on natural images. Nevertheless, its effectiveness decreases when applied directly to plant disease segmentation. To address the challenge, several SAMbased adaptation methods have been proposed. SAM-IE (Bello et al., 2025) integrates SAM with an image enhancement module, achieving notable accuracy gains via ResNet50. Madadum et al (Madadum et al., 2025). combines SAM with YOLO architectures and proposes an augmentation strategy to mitigate class imbalance, where SAM extracts object masks from rare classes and pastes them onto field backgrounds, leading to improved performance. SAM-YOLOv8 (Zhao et al., 2025b) has been developed for automated leaf segmentation and annotation, using SAM to generate training labels offline and YOLOv8 for real-time inference. However, beyond the domain gap between generic natural-image data and agricultural imagery (Ding et al., 2022), a more critical issue is that SAM primarily captures global structural cues and is less sensitive to subtle local texture differences between lesions and visually similar structures such as leaf veins. **Figure 2** shows that the fine-tuned SAM focuses more on global shape. As a result, it may miss small or early-stage lesions and often produces coarse or inaccurate lesion boundaries. Another line of models which combine SAM with CNN branch has appeared. SAMUS (Lin et al., 2024) adapts SAM introduces a parallel CNN branch with cross-branch attention and an auto prompt generator, enabling end-to-end automatic segmentation while preserving SAM’s global modeling strength. EMSAM (Li et al., 2025) enhances SAM with a multi-scale adaptive adapter and a local feature extraction module to capture blurred boundaries and fine-grained lesion details. Despite these advances, existing SAM-based methods for plant disease segmentation share a common limitation that they fuse global SAM features with local CNN features using deterministic strategies, without explicitly modeling the reliability or uncertainty of each feature source. In complex field environments, the confidence of global structural cues and local texture details can vary substantially across regions. Fusing all features indiscriminately often propagates noise and leads to unsatisfactory segmentation performance.

To address these limitations of SAM and SAM variants in plant disease segmentation, we enhance it by incorporating complementary local lesion cues and reliability-aware feature fusion. Specifically, a ResNet50 branch is used to extract fine-grained lesion textures and boundary details that are difficult for SAM to capture. These detail features are fused with the encoder representations of SAM and then fed into the SAM decoder, allowing the model to preserve SAM’s strong global structural perception while improving sensitivity to subtle lesion patterns. However, because SAM features mainly encode global structural cues, whereas local detail features provide fine-grained lesion textures and refined boundaries, directly fusing the two feature streams may not fully exploit their complementary strengths. More importantly, the major challenge lies in the integration of heterogeneous representations under complex agricultural environments. Existing fusion strategies, such as direct addition, concatenation, or similarity-based attention, generally treat features equally and lack explicit reliability modeling. Consequently, unreliable responses caused by cluttered backgrounds, illumination variations, and vein interference may dominate the fusion process and degrade lesion segmentation quality. To address this issue, we propose a reliability-guided variational fusion framework that explicitly models feature uncertainty and dynamically prioritizes reliable lesion representations during cross-branch fusion. Specifically, an uncertainty encoder estimates the confidence of branch features through probabilistic parameterization, and a variational fusion module adaptively emphasizes reliable lesion cues while suppressing interfering responses from cluttered backgrounds. In addition, Kullback–Leibler divergence regularization is used to stabilize cross-feature alignment during training. Through these designs, the proposed method improves the segmentation of small lesions and ambiguous boundaries in real agricultural environments.

Overall, the main contributions of this work can be summarized as follows:

- We improve SAM for plant disease segmentation by introducing a ResNet-50-based detail compensation branch that supplements the fine-grained lesion textures and boundary cues that are difficult for SAM to capture, thereby improving segmentation in complex field scenes.
- We propose a novel reliability-guided feature fusion strategy to integrate heterogeneous global structural cues from SAM with fine-grained lesion information from the detail compensation branch. This is achieved through an uncertainty-aware variational framework by using probabilistic confidence estimation to dynamically weight features, rather than simple attention or concatenation. And KullbackLeibler divergence regularization is introduced to help stabilize feature alignment and improve fusion robustness.
- Extensive experiments on three real-world datasets, namely PlantSeg (Wei et al., 2026), PlantDoc-Seg (Singh et al., 2020), and ATLDSD (Feng and Chao, 2022), demonstrate that the proposed method consistently outperforms state-of-the-art approaches, achieving DSC scores of 81.05%, 91.12%, and 88.27%, respectively.

## 2 Materials and methods

### 2.1 Dataset description

To evaluate the effectiveness of the proposed method for plant disease segmentation, experiments were conducted on three publicly available datasets: PlantSeg (Wei et al., 2026), PlantDoc-Seg (Singh et al., 2020), and ATLDSD (Feng and Chao, 2022). These datasets cover diverse plant species, disease categories, and imaging conditions, thereby providing a comprehensive benchmark for performance assessment.

#### 2.1.1 PlantSeg dataset

PlantSeg is a large-scale in-the-wild benchmark for plant disease segmentation, designed to address the main limitations of existing datasets, including insufficient annotation accuracy, restricted acquisition scenarios, and limited dataset scale. It contains 11,458 images with fine-grained disease segmentation masks, covering 34 plant species of considerable economic and nutritional value and 115 common plant diseases. Specifically, it includes 45 vegetable diseases, 39 fruit diseases, 9 cash crop diseases, and 22 staple crop diseases, thereby covering the major crop categories in agricultural production.

Unlike datasets acquired under controlled laboratory conditions, all images in PlantSeg were collected from real-world field environments, exhibiting complex backgrounds, varying viewpoints, and significant illumination changes. Some representative samples from PlantSeg are shown in **Table 1**.

**Table 1:** Representative images of PlantSeg used in this study.

Species	Apple	Tomato	Banana	Citrus	Grape	Wheat
Disease	black rot	late blight	panama disease	canker	spot	loose smut
Sample ID	apple_ black rot_ 38	tomato, late blight. 66	banana_ panama disease_ 31	citrus canker 44	grape_ leaf spot_ 41	wheat loose smut_ Google_ 0206
Picture	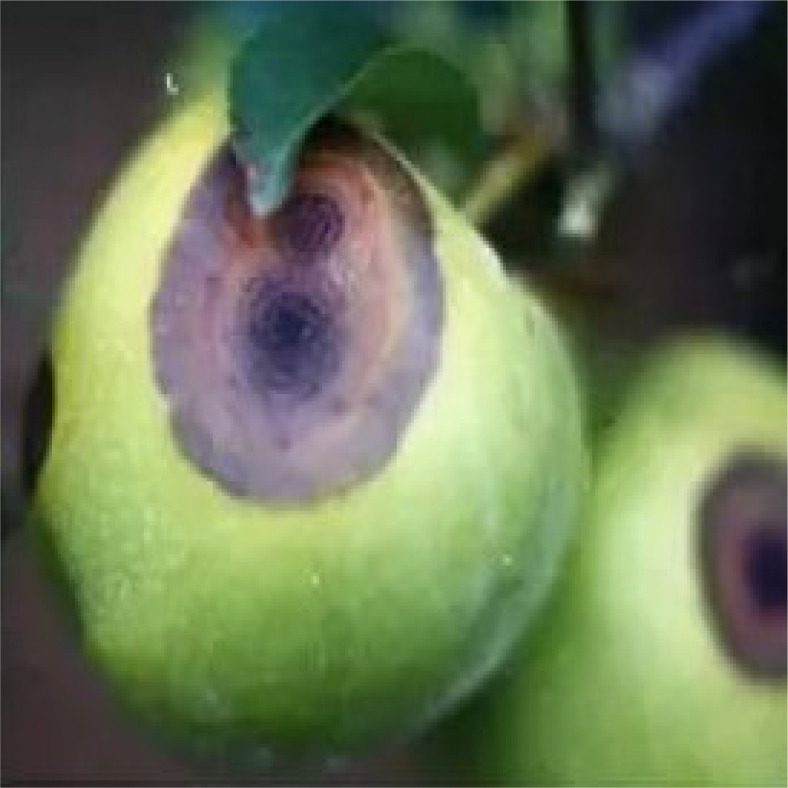	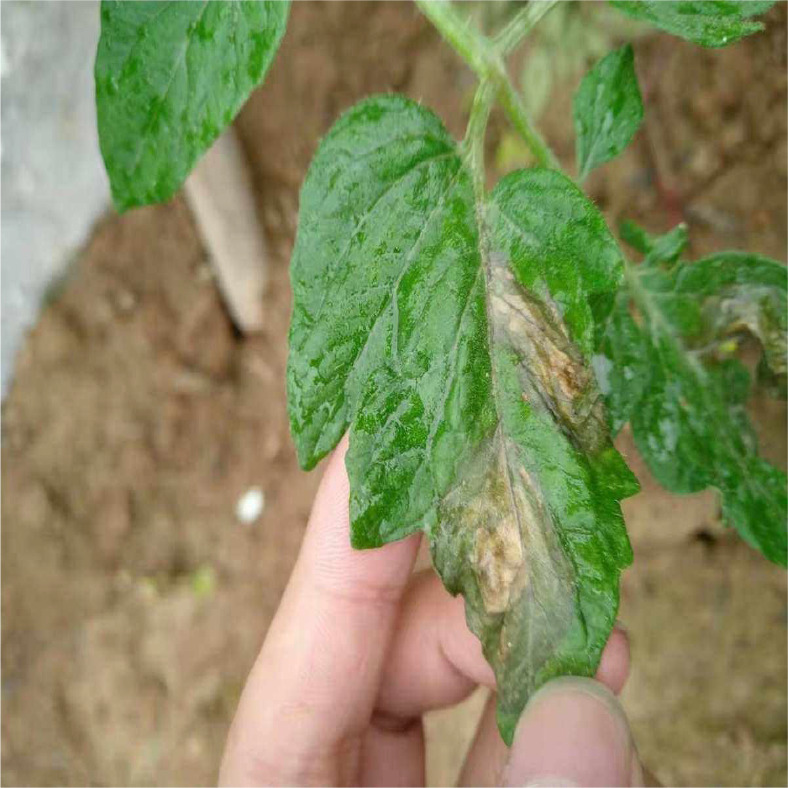	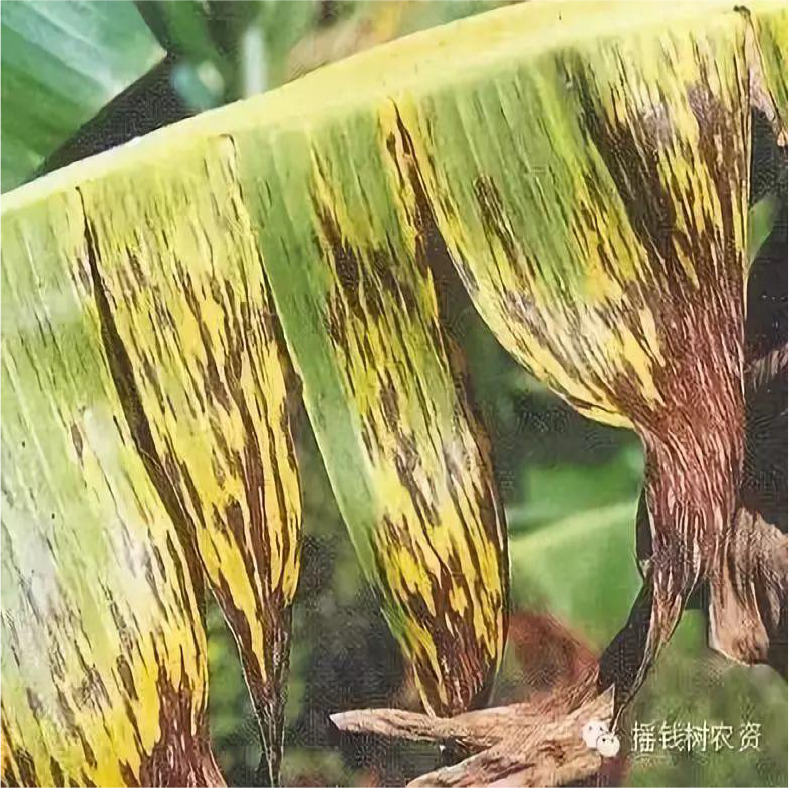	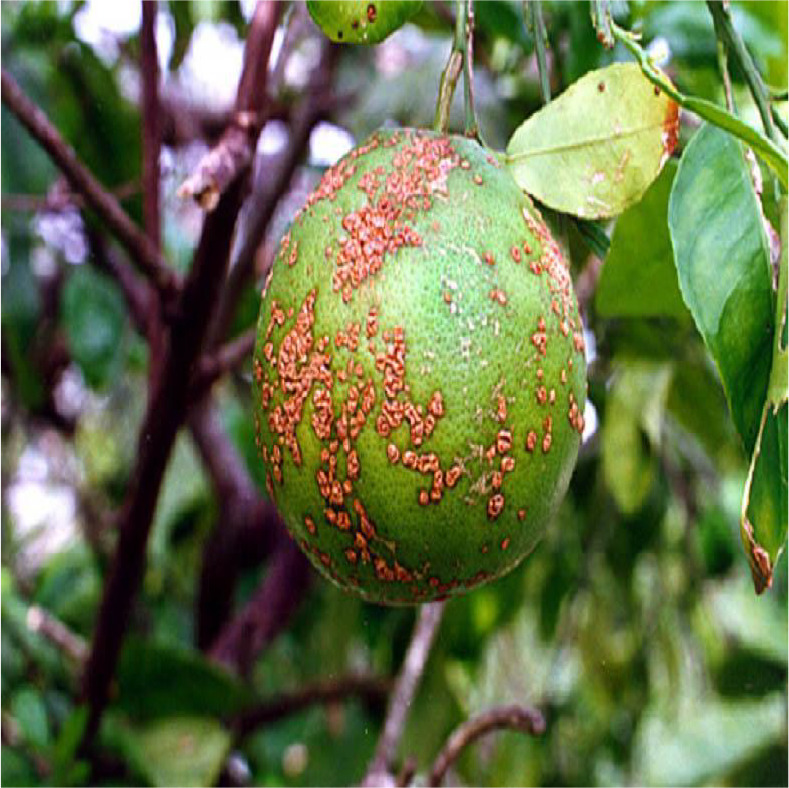	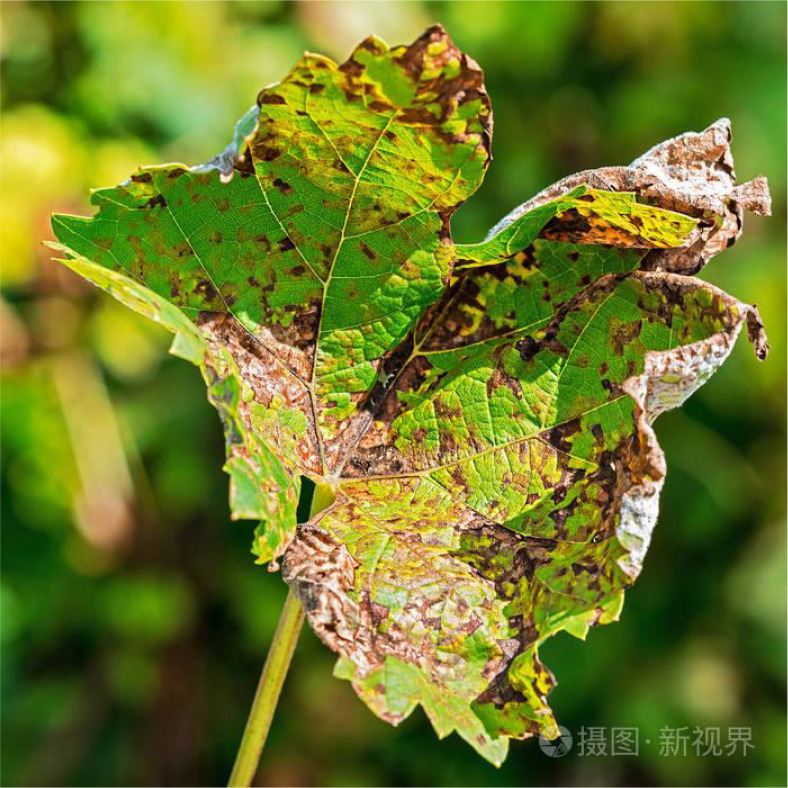	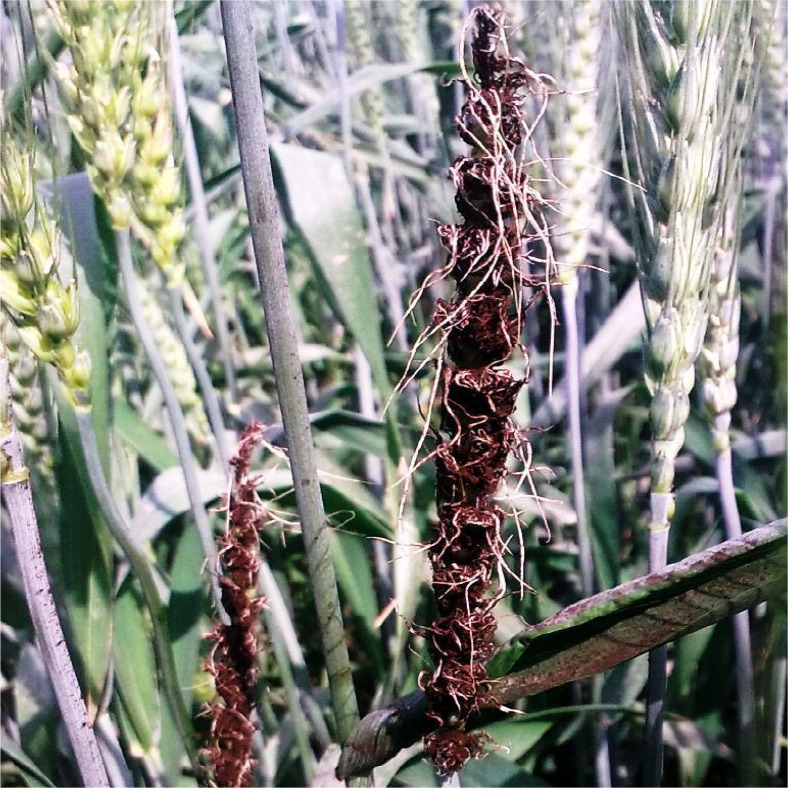

#### 2.1.2 PlantDoc-Seg dataset

PlantDoc was developed to address the scarcity of image data in large-scale field-based agricultural scenarios and to support the development and practical application of computer vision techniques for early crop disease detection. It has high practical value for agricultural computer vision research. The dataset contains 2,598 high-quality images with professional annotations, covering 13 economically important crops, including tomato, corn, banana, and cotton. Some representative samples from PlantDoc are shown in **Table 2**.

**Table 2:** Representative images of PlantDoc-Seg used in this study.

Species	Apple	Bell pepper	Corn	Grape	Tomato	Tomato
Disease	rust disease	spot	rust	Black rot	Septoria spot	bacterial spot
Sample ID	apple-rust disease_10 845384767	Bacterial spot symptoms on-pepper leaves-for web	**Figure 3**_Common_rust	8–21 Anth racnose shotholing ANNE MIEK	6447731_orig	10–3 bacterial speck-on tomato RON
Picture	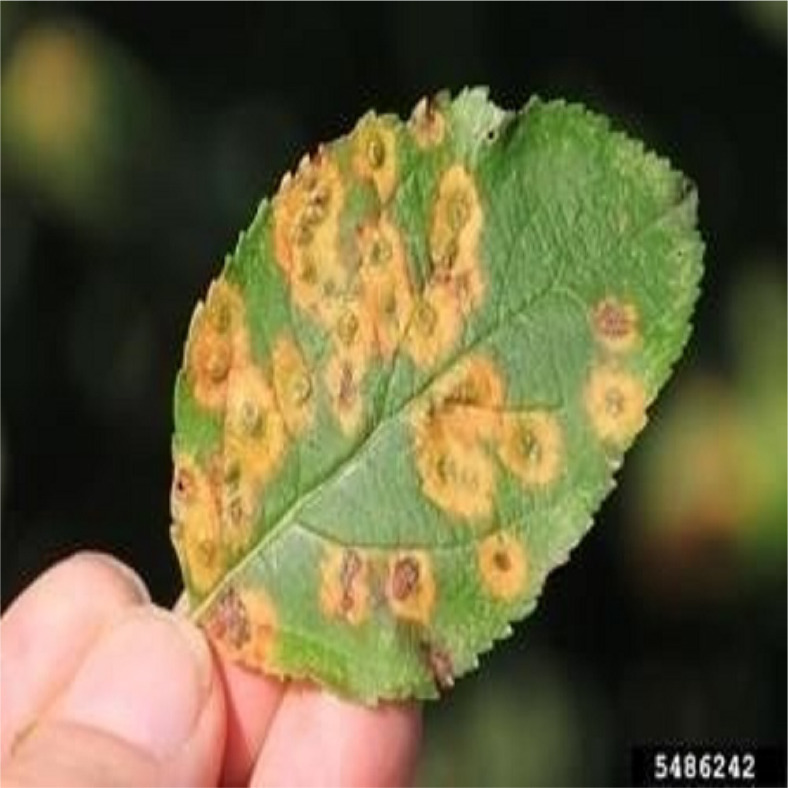	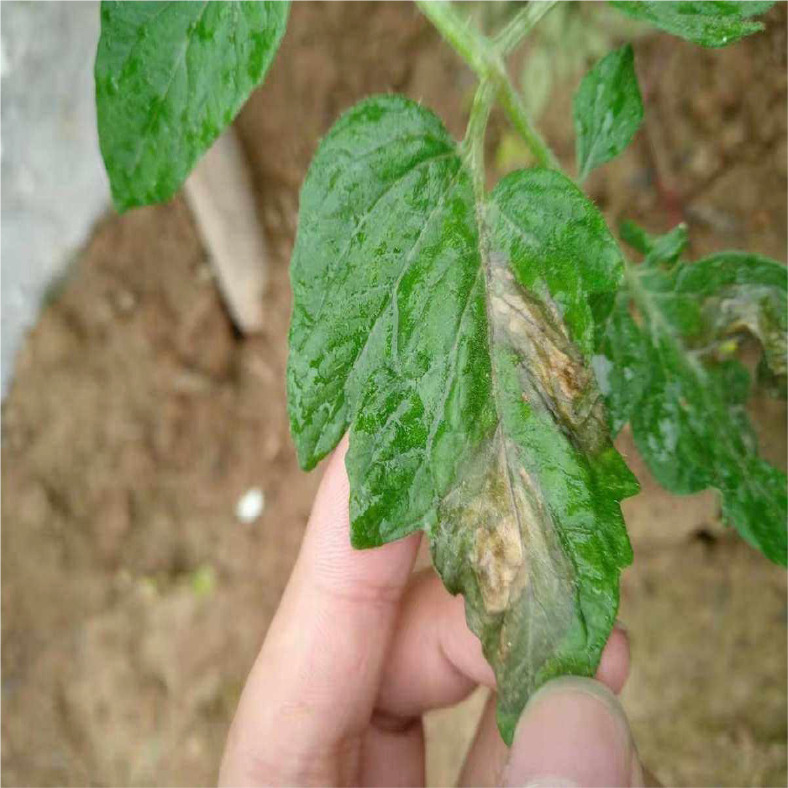	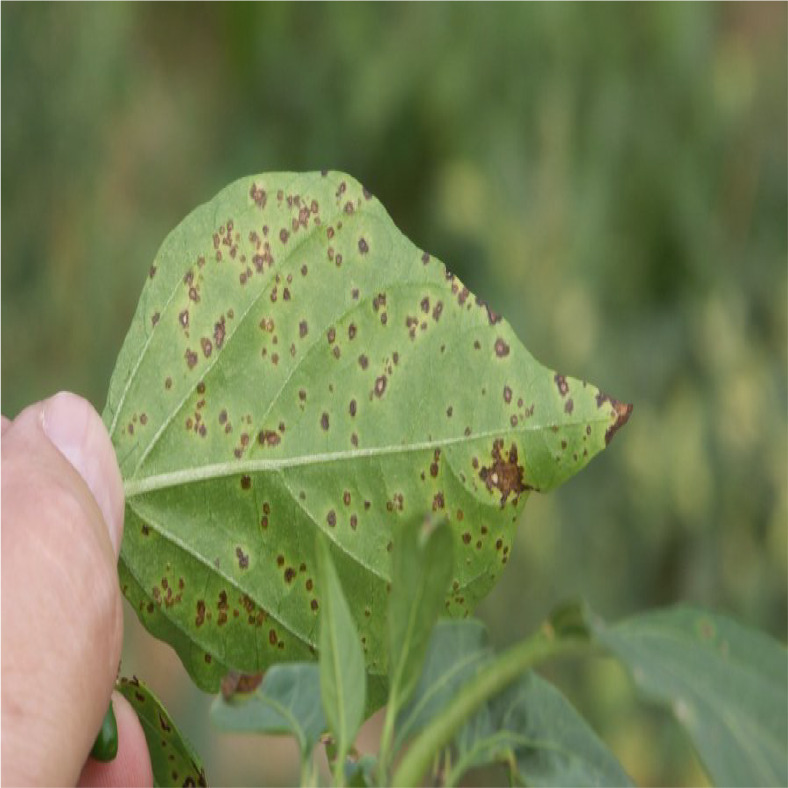	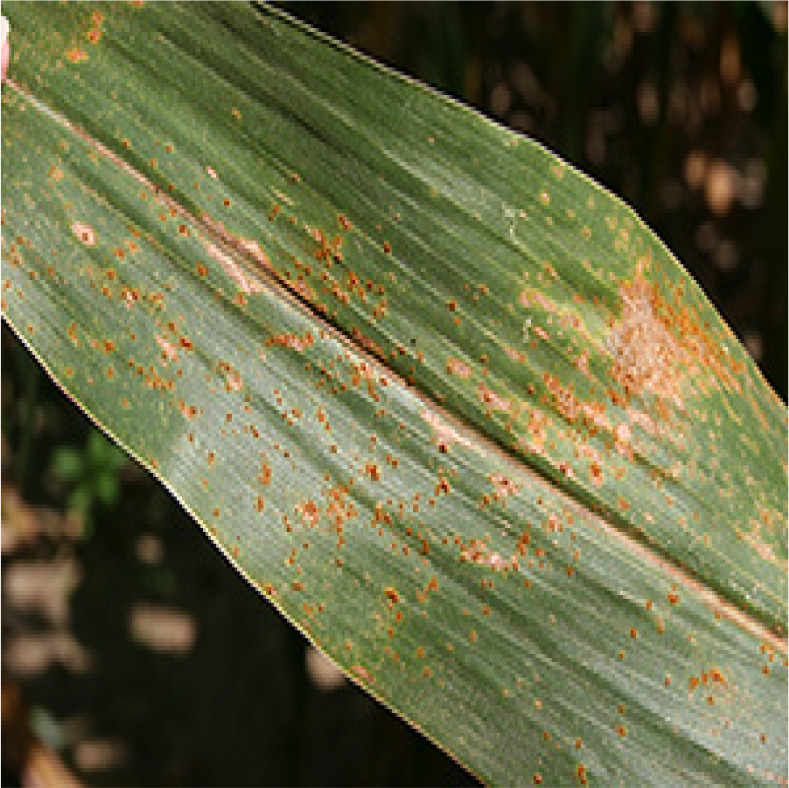	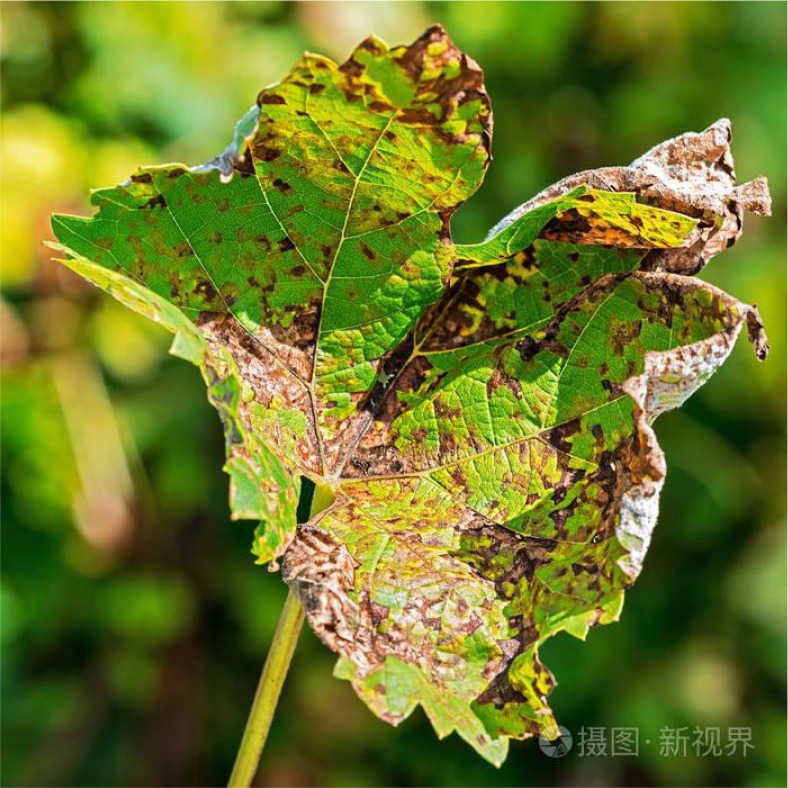	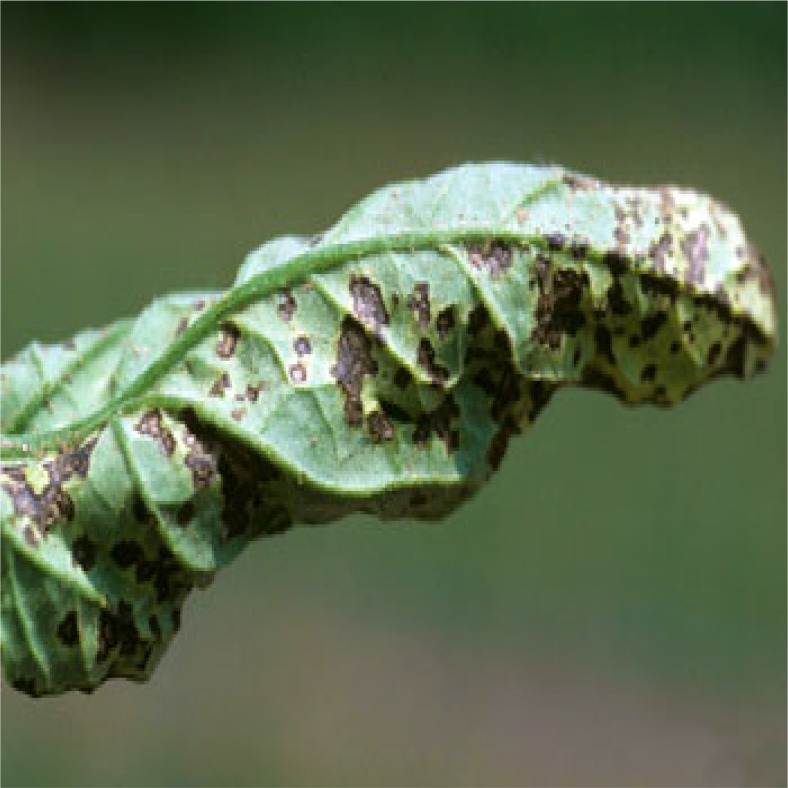

However, the original PlantDoc dataset is mainly designed for plant disease detection and recognition, and it does not provide pixel-level segmentation masks. Therefore, we used a community-provided segmentation subset from Kaggle^^1^^, which we refer to as PlantDoc-Seg in this study. This subset is derived from PlantDoc and contains 588 diseased leaf images with corresponding binary masks, enabling supervised leaf disease segmentation.

#### 2.1.3 ATLDSD dataset

The Apple Tree Leaf Disease Segmentation Dataset (ATLDSD) is a dedicated dataset for the semantic segmentation of apple leaf diseases. It provides a high-quality data foundation for the early screening, accurate segmentation, and severity diagnosis of apple diseases. The dataset contains 1,644 original images, covering four common types of apple leaf diseases, namely alternaria leaf spot, gray spot, brown spot, and rust, as well as healthy leaves.

The images were collected under diverse conditions, including different weather conditions and times of day, and their validity was verified by agricultural experts. Characterized by scene authenticity, annotation accuracy, and task adaptability, ATLDSD provides important data support for research on apple leaf disease segmentation and serves as a reference for the development of disease monitoring technologies for other crops in smart agriculture. Some representative samples from ATLDSD are shown in **Table 3**.

**Table 3:** Representative images of ATLDSD used in this study.

Disease	Alternaria leaf spot	Brown spot	Gray rust	Healthy	Rust
Sample ID	00416	001019	003023	IMG 20190726_191205	004161
Picture	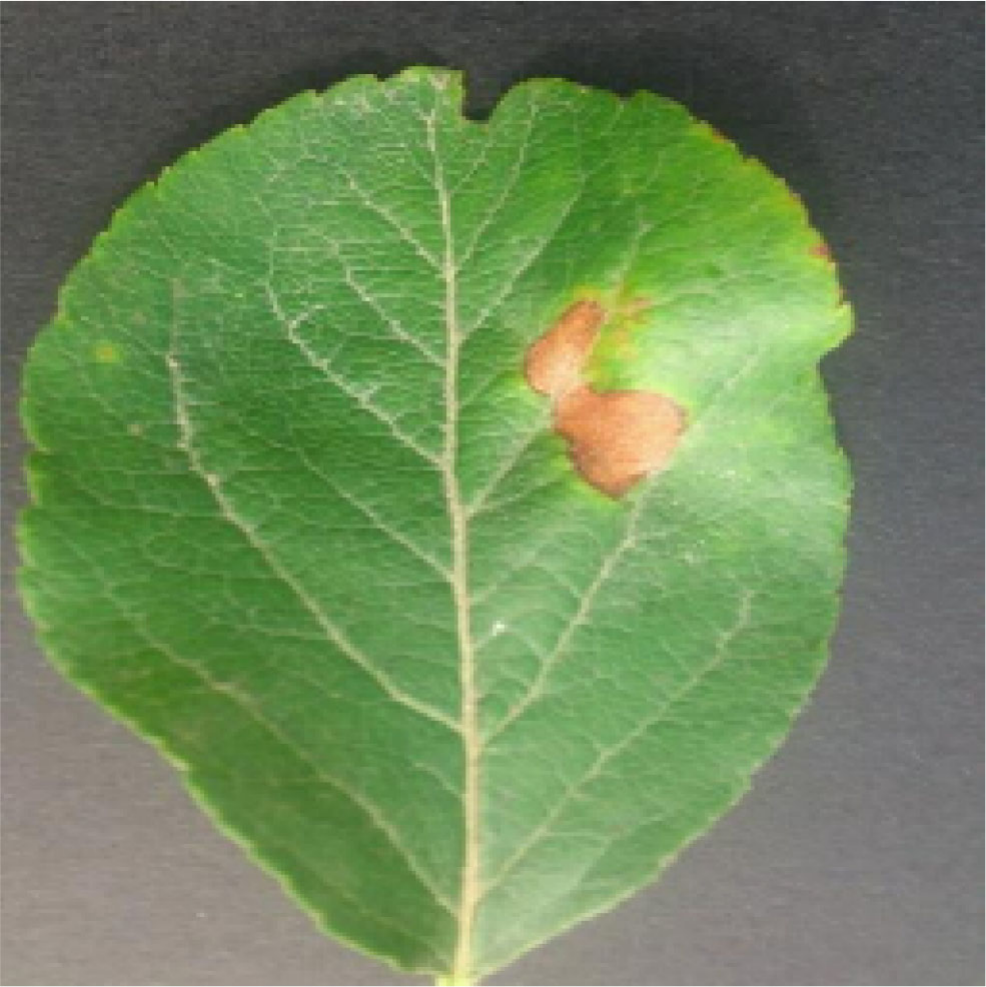	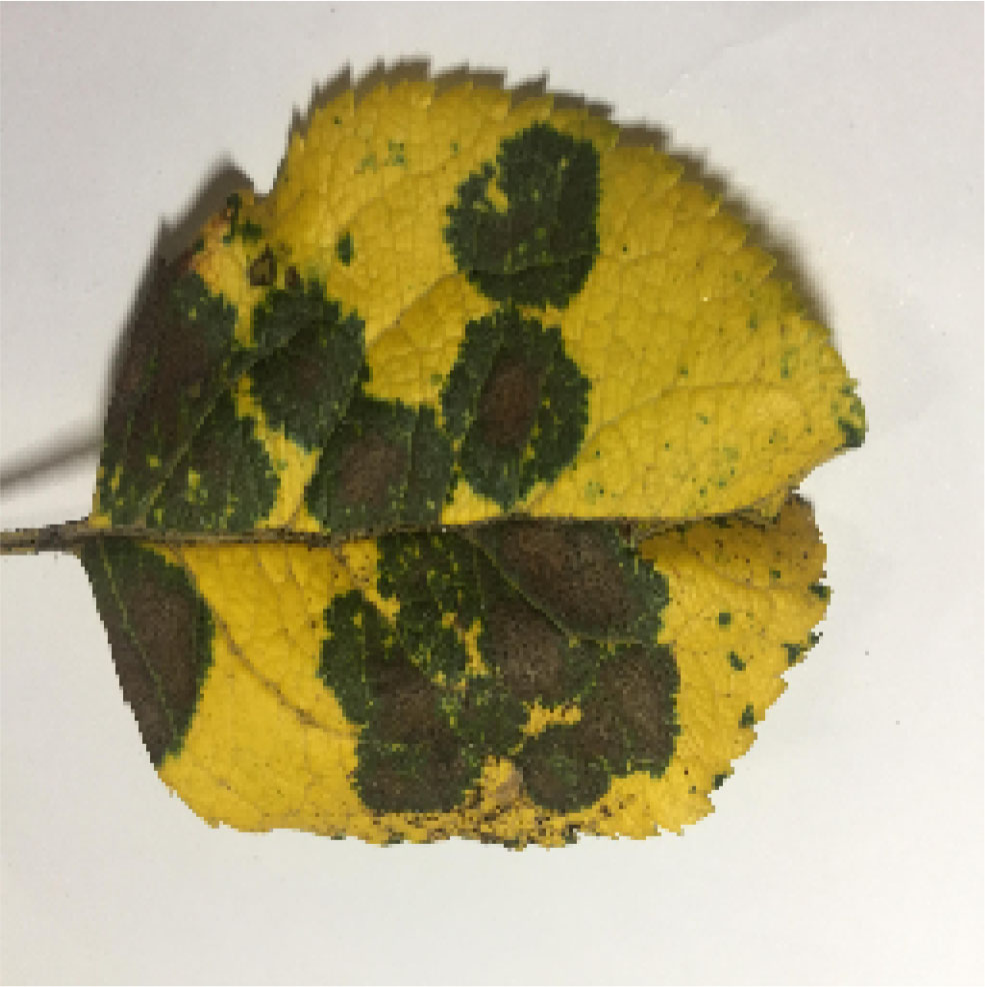	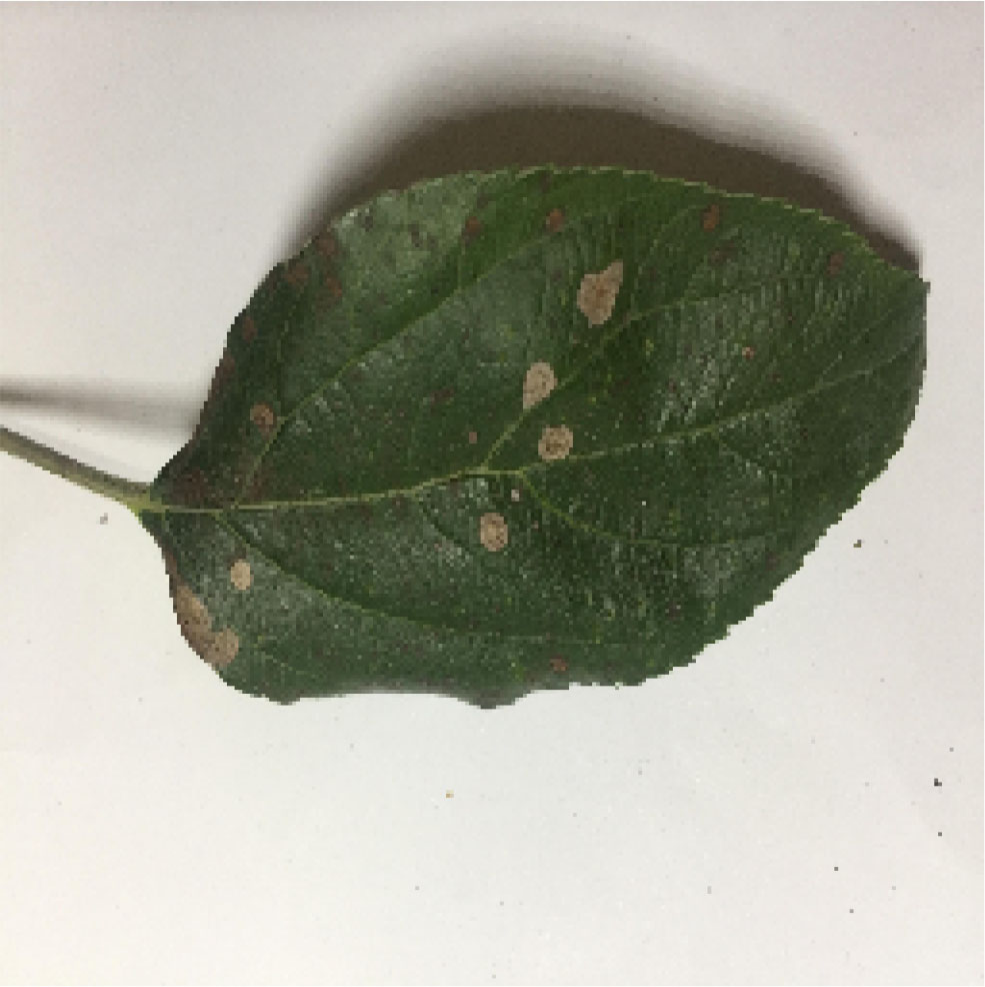	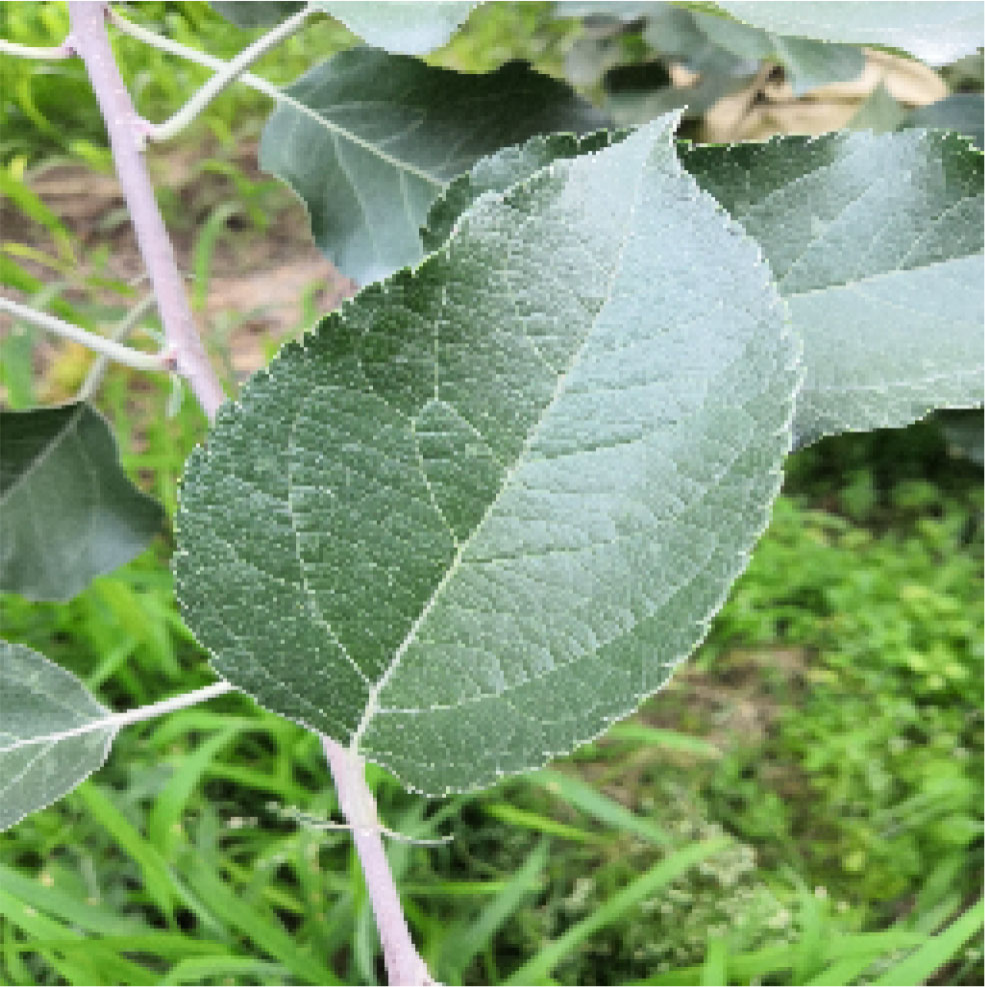	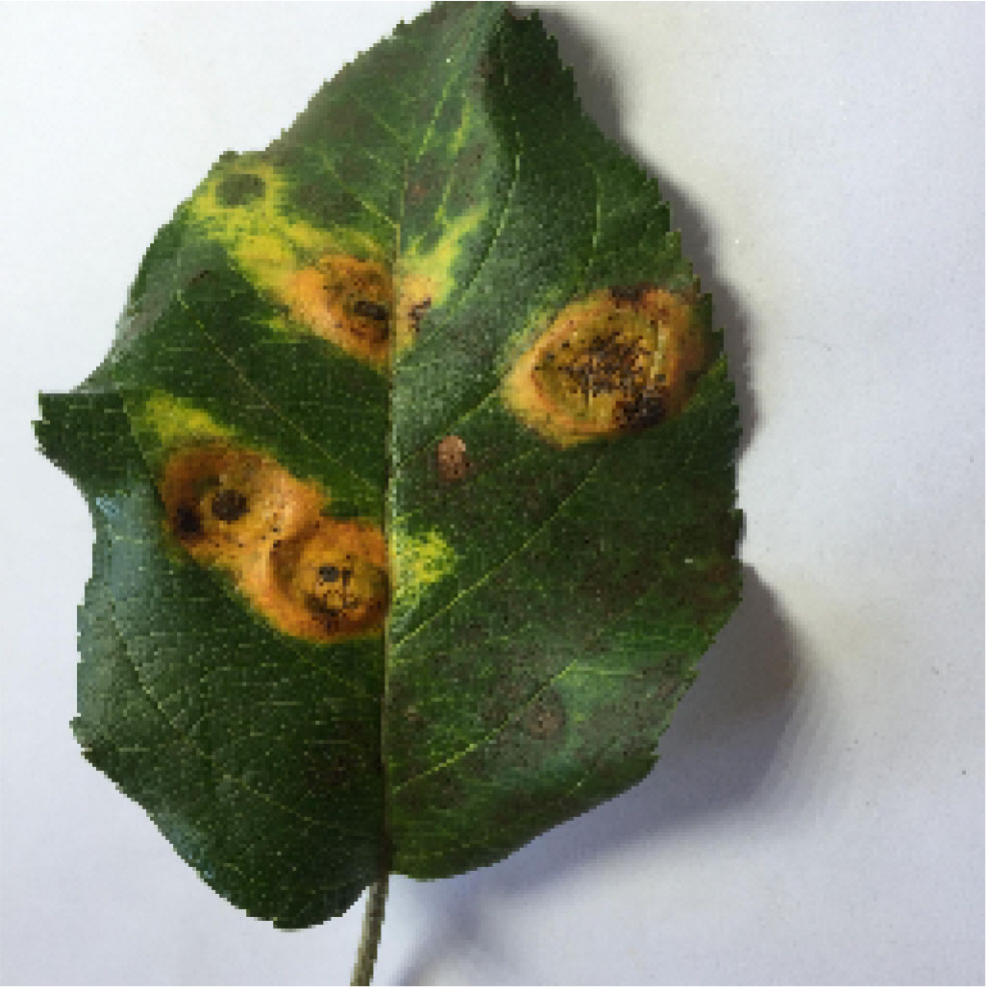

### 2.2 ReLeaf-SAM plant disease segmentation model

**Figure 3:**
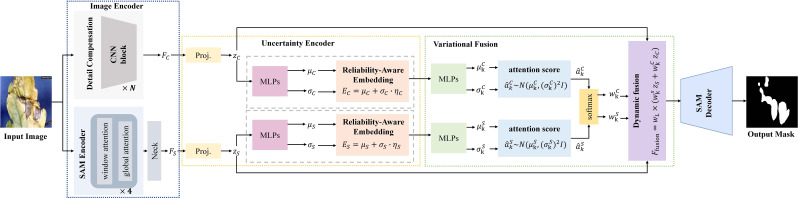
Architecture overview of the proposed method. The framework comprises a global modeling branch and a detail compensation branch for extracting complementary features, an uncertainty encoder that projects and models these features based on uncertainty, and a variational fusion module for dynamic feature integration. The decoder is implemented using the SAM mask decoder.

As shown in **Figure 3**, the proposed architecture adopts SAM as the base segmentation model to achieve accurate localization of overall lesion regions. Meanwhile, ResNet-50 is introduced to extract fine-grained features and compensate for SAM’s limited sensitivity to subtle texture differences between lesions and plant veins by leveraging its multi-layer convolutional and residual structures. To address representational heterogeneity and unreliable feature fusion in the global-detail collaborative architecture, the model replaces traditional linear fusion methods with an uncertainty-aware fusion strategy consisting of an uncertainty encoder and a variational fusion module. Specifically, the uncertainty encoder models the extracted features from the global and compensation branches as probability distributions in latent spaces, enabling uncertainty estimation for different feature components through element-wise calibration. Based on the resulting distribution parameters, the variational fusion module dynamically calculates weights, assigning higher weights to features with lower uncertainty and higher reliability while suppressing interfering features. In addition, Kullback–Leibler (KL) divergence regularization is imposed to encourage the feature distributions and their attention weights to align with a standard normal distribution, thereby reducing distributional fluctuations caused by complex environments and stabilizing cross-branch feature alignment.

#### 2.2.1 Global modeling branch and detail compensation branch

The image encoder serves as the core feature extraction unit of the proposed segmentation framework. Through the collaboration of the global modeling branch and the detail compensation branch, the network captures shape-aware, category-agnostic features as well as discriminative local texture features, thereby providing comprehensive and complementary representations for subsequent feature fusion.

For the global modeling branch, we adopt SAM as the base model because of its strong global semantic modeling capability and transferability. Built upon the Vision Transformer (ViT) architecture with selfattention and multi-scale feature extraction, SAM effectively captures long-range dependencies and global contextual information. In addition, its large-scale pre-training endows it with strong generalization ability for new segmentation tasks. The feature map *F_S_*produced by this branch mainly characterizes the global shape priors of target regions, thereby providing a reliable basis for overall lesion localization.

To compensate for the limitation of the SAM branch in local detail modeling, ResNet-50 is introduced as the detail compensation branch to extract fine-grained features through its deep convolutional and residual structures. By preserving local spatial details while enhancing feature representation, this branch effectively captures subtle lesion textures and boundary information. The feature map *F_C_*produced by this branch focuses on detailed target information, which helps refine the coarse localization results of the SAM branch and improves segmentation boundary accuracy and regional completeness.

#### 2.2.2 Uncertainty encoder

**Figure 4:**
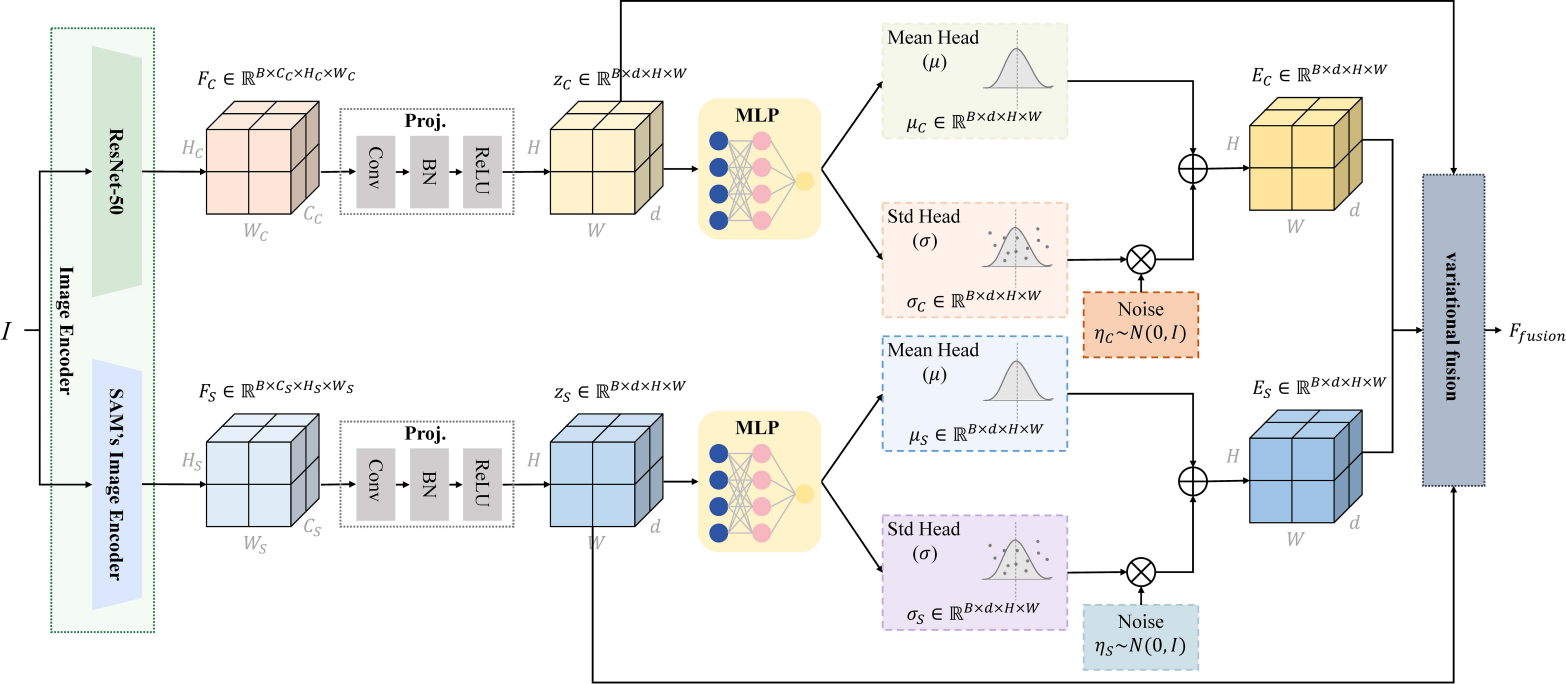
Structure of the uncertainty encoder. It conducts probabilistic representation of the projected features based on the Gaussian distribution and ultimately generates embedding features integrated with uncertainty.

To address the issue of reliability fluctuations in features from the global modeling branch and detail compensation branch under complex plant disease scenarios, this study introduces an uncertainty encoder that quantifies the inherent uncertainty of features through probabilistic modeling, thereby providing a reliable confidence basis for variational fusion. The core objective of the uncertainty encoder is to calibrate the pre-extracted features *F_S_*and *F_C_*by modeling their uncertainties, suppress the interference of low-reliability features, and enhance the purity of feature representations. The overview architecture of the uncertainty encoder is shown in **Figure 4**.

The uncertainty encoder adopts a diagonal Gaussian latent distribution (Barndorff-Nielsen, 1997) for unified modeling of the two-branch features, mapping the SAM branch feature *F_S_*and the ResNet-50 branch feature *F_C_*into probability distributions containing confidence information, respectively. To ensure the dimensional consistency of *F_C_*and *F_S_*, we obtain *z_C_*and *z_S_*through dimension mapping, thereby guaranteeing that the two-branch features lie in the same feature space. Specifically, for the 512×512 input, the compensation branch feature (2048×16×16) is projected to 768×32×32 using ConvTranspose2d(2048, 768, 2, 2) followed by BN and ReLU, and the SAM branch feature (768×32×32) is projected by conv1×1 followed by BN and Relu. The resulting maps *z_C_*and *z_S_*are both 768 × 32 × 32. The uncertainty encoder models *z_C_*and *z_S_*as diagonal Gaussian latent distributions (Equations 1–4):

(1) $$P(E_{S}|z_{S})=N(E_{S};\mu_{S},{\left(\sigma_{S}\right)}^{2}I)$$

(2) $$\mu_{S},\sigma_{S}=MLP\left(z_{S}\right)$$

(3) $$P\left(E_{C}|z_{C}\right)=N\left(E_{C};\mu_{C},{\left(\sigma_{C}\right)}^{2}I\right)$$

(4) $$\mu_{C},\sigma_{C}=MLP\left(z_{C}\right)$$

For each branch feature, a lightweight MLP network is used to learn the mean *µ_C_* and *µ_S_* and the standard deviation *σ_C_* and *σ_S_* of the Gaussian distribution. The MLP adopts a two-layer fully connected structure, which can be referred as *Linear* (*C,C/*2) → *BN* → *ReLU* → *Linear* (*C/*2*,C*). Specifically, the MLPs that predict the mean *µ_C_* and *µ_S_* directly output the result of the second layer, while the outputs of the MLPs that predict the standard deviation *σ_C_* and *σ_S_* are transformed via exp(0.5 · *x*) after the second layer to ensure positivity. *µ_C_* and *µ_S_* represent the core semantic information of the features, and *σ_C_* and *σ_S_* quantify the uncertainty of the corresponding feature dimensions. A larger value of *σ_C_* or *σ_S_* indicates that the feature in this region is more strongly affected by factors such as background interference and illumination variation, resulting in lower reliability.

To ensure differentiability during end-to-end model training, the reparameterization trick is employed to generate stochastic embedding features as Equations 5–6:

(5) $$E_{S}=\mu_{S}+\sigma_{S}\eta_{S}$$

(6) $$E_{C}=\mu_{C}+\sigma_{C}\eta_{C}$$

where the standard Gaussian noise $\eta_{S},\eta_{C}∼N\left(0,I\right)$. This operation encodes uncertainty information into the feature embeddings, enabling *E_S_* and *E_C_* to simultaneously carry semantic representation and reliability measurement information.

#### 2.2.3 Variational Fusion

**Figure 5:**
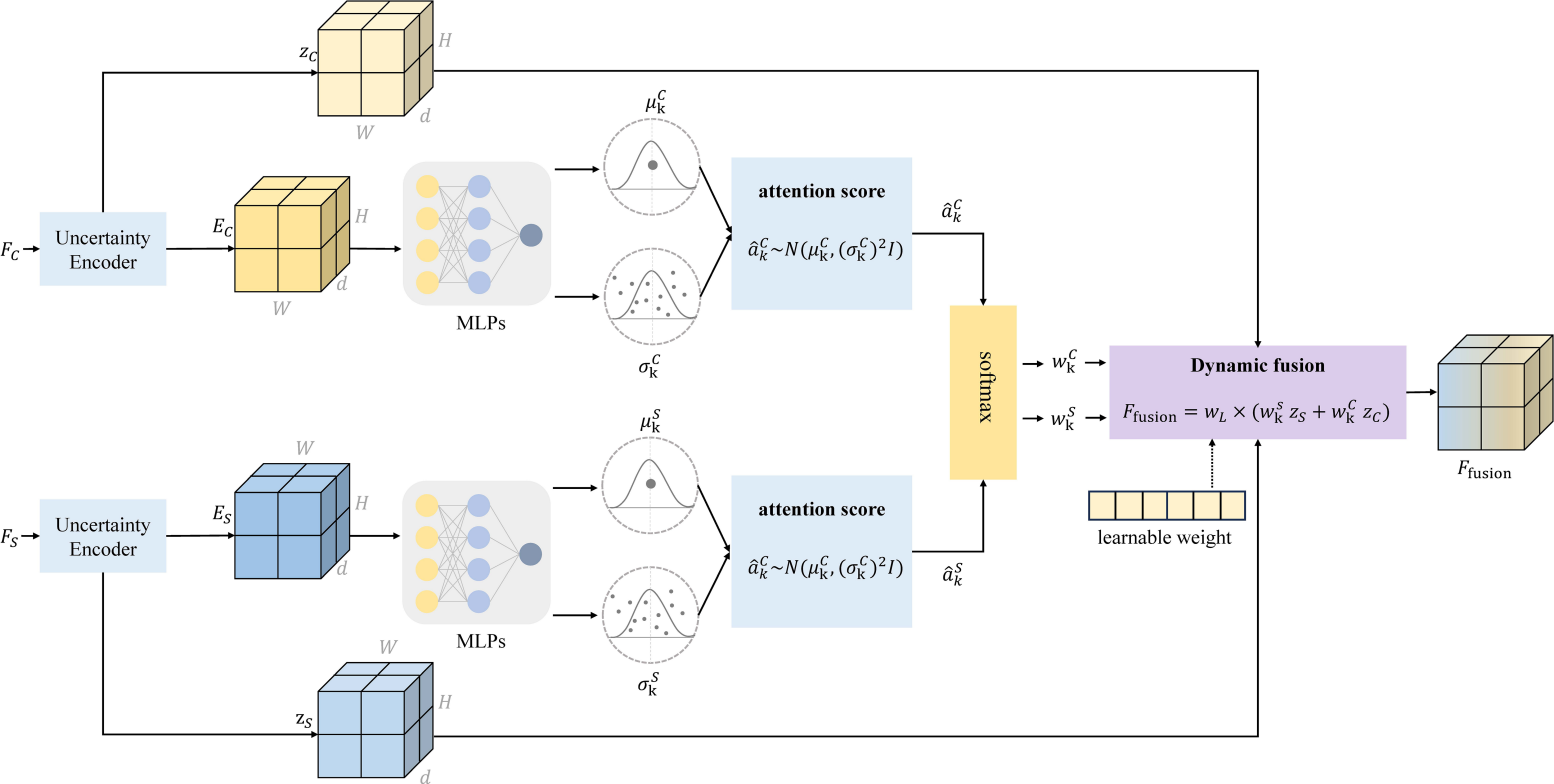
Structure of the variational fusion module. It calculates attention scores based on the Gaussian distribution and fuses the complementary features through a dynamic mechanism.

As the core fusion unit of our architecture, the variational fusion module is designed to address the representational heterogeneity between the global semantic features from the SAM branch and the local fine-grained features from the ResNet-50 branch, thereby achieving adaptive and highly reliable fusion. In contrast to traditional linear fusion methods such as element-wise addition and channel concatenation, as well as attention fusion approaches relying on feature similarity, this module dynamically assigns fusion weights using feature probability distribution parameters as the core basis. By quantifying feature uncertainty, it prioritizes reliable features while suppressing interfering information, thereby providing purer and more robust feature representations for subsequent plant disease segmentation tasks. The overview architecture of variational fusion is shown in **Figure 5**.

The input to this module consists of the reliability-aware feature embeddings *E_S_* and *E_C_* produced by the uncertainty encoder. The probability distribution parameters of attention scores are predicted via separate lightweight MLPs. blueSimilar to the MLPs in the uncertainty encoder, the MLPs adopt a (Linear(C,C/2)→BN→ReLU→Linear(C/2,1)) structure, and the same activation conventions as the uncertainty encoder for the mean and the standard deviation branch. For *E_S_* and *E_C_*, the MLP outputs the mean $\mu_{k}^{S}$, $\mu_{k}^{C}$ and standard deviation $\sigma_{k}^{S}$, $\sigma_{k}^{C}$ of the attention scores. Different from traditional attention mechanisms that directly output deterministic scores, the variational fusion module samples the attention score ${\hat{a}}_{k}^{S}$ and ${\hat{a}}_{k}^{C}$ from the corresponding normal distributions as Equations 7–8:

(7) $${\hat{a}}_{k}^{S}∼N(\mu_{k}^{S},{\left(\sigma_{k}^{S}\right)}^{2}I)$$

(8) $${\hat{a}}_{k}^{C}∼N(\mu_{k}^{C},{\left(\sigma_{k}^{C}\right)}^{2}I)$$

To achieve reasonable weight assignment for features, ${\hat{a}}_{k}^{S}$ and ${\hat{a}}_{k}^{C}$ are normalized using the softmax function to obtain the base fusion weights $w_{k}^{S}$ and $w_{k}^{C}$. Considering the requirement for dynamic balance between global semantic localization and local detail delineation in plant disease segmentation tasks, a learnable scalar parameter *w_L_* is introduced into the module to perform secondary modulation on the final fused features *F_fusion_* as Equation 9.

(9) $$F_{fusion}=w_{L}\left(w_{k}^{S}z_{S}+w_{k}^{C}z_{C}\right)$$

### 2.3 Loss function

#### 2.3.1 Kullback-Leibler Divergence

Kullback-Leibler (KL) Divergence (Ji et al., 2022) is a fundamental metric for measuring the discrepancy between two probability distributions. Its core role is to regularize the learned feature distributions by measuring their deviation from a target prior. In segmentation tasks, KL Divergence is commonly used to guide the latent feature distributions toward a predefined stable prior, thereby reducing the uncertainty caused by distributional fluctuations. Mathematically, it computes the expected value of the log-likelihood ratio between two distributions. During training, minimizing this term encourages the learned feature distributions to approach the target prior, thereby improving the stability of feature learning, enhancing the generalization ability of the model, and providing a reliable basis for subsequent feature fusion.

In our study, KL divergence is integrated into the fusion module to enhance feature reliability. Specifically, KL regularization is imposed on two types of key distributions. The first is the latent feature distributions of the SAM branch *E_S_* and the ResNet-50 branch *E_C_* generated by the uncertainty encoder. The second is the distributions of the two-branch attention logits ${\hat{a}}_{k}^{S}$ and ${\hat{a}}_{k}^{C}$. KL divergence measures the discrepancy between these distributions and the standard normal distribution N(0,I), which is formulated as Equation 10:

(10) $$KL\left(N\left(\mu ,\sigma^{2}I\right)∥N\left(0,I\right)\right)=\frac{1}{2}\sum_{d=1}^{D}\left(\sigma_{d}^{2}+\mu_{d}^{2}-\text{log }(\sigma_{d}^{2})-1\right)$$

where *D* denotes the dimension of features or attention weights, while *μ_d_* and σ*_d_* represent the mean and standard deviation of the *d*-th dimension, respectively. Following Equation 11, the total KL loss *L_KL_* is obtained by summing the KL terms of $L_{E_{S}}$, $L_{E_{C}}$, $L_{{\hat{a}}_{k}^{S}}$ and $L_{{\hat{a}}_{k}^{C}}$, thereby effectively suppressing the influence of unreliable features and reducing distributional fluctuations caused by cross-domain shifts.

(11) $$L_{KL}=L_{E_{S}}+L_{E_{C}}+L_{{\hat{a}}_{k}^{S}}+L_{{\hat{a}}_{k}^{C}}$$

#### 2.3.2 Final Loss Function

We incorporate the cross-entropy loss *L_ce_* and Dice loss *L_Dice_* (Li et al., 2020) into the final loss function. Specifically, *L_ce_* focuses on pixel-level classification accuracy. By minimizing the logarithmic discrepancy between predicted probabilities and ground truth labels, it enhances the pixel discriminability between lesion regions and background, facilitating sharper segmentation boundaries and improving segmentation reliability. On the other hand, *L_Dice_* is designed to alleviate class imbalance issues, particularly in the presence of small targets and sparse lesion regions. It measures the overlap between the predicted masks and the ground truth, thereby emphasizing the integrity of target regions and reducing missed segmentation or fgmented segmentation of small lesions caused by sample imbalance. The final loss function *L* is defined as Equation 12:

(12) $$L=L_{KL}+L_{Dice}+L_{ce}$$

## 3 Results and analysis

### 3.1 Experimental environment

All experiments were implemented based on PyTorch 2.1.0 and conducted on a hardware platform equipped with 8 × NVIDIA GeForce RTX 4090 GPUs (24 GB memory per card) and 128 GB system RAM, with the Ubuntu 20.04.6 LTS operating system. We adopted the pre-trained SAM model (Kirillov et al., 2023) with ViT-B as the backbone and ResNet-50 (He et al., 2016) as the detail compensation branch. The model was trained for 300 epochs using AdamW (Loshchilov and Hutter, 2019) with an initial learning rate of 0.001. Following nnUNet (Isensee et al., 2021), *L_KL_*, *L_Dice_*and *L_ce_*of the loss function *L* are added using fixed equal weights. The training batch size was set to 16. Data augmentation strategies included horizontal, vertical flipping and random rotation. Detailed hardware and software configurations are summarized in **Table 4**.

**Table 4:** Experimental environment configuration.

Environment	Configuration
Python Version	3.11.11
Operating System	Ubuntu 20.04.6 LTS
GPU	NVIDIA GeForce RTX 4090 × 8
GPU Memory	24 GB per GPU
System Memory	128 GB
Framework	PyTorch 2.1.0
Input Image Size	512 × 512 pixels
Number of Training Epochs	300
Batch Size	16
Optimizer	AdamW
Initial Learning Rate	0.001

### 3.2 Evaluation metrics

In this study, the performance of the proposed segmentation model was quantitatively evaluated using three core metrics: Dice Similarity Coefficient (DSC), Intersection over Union (IoU), Precision (P), and Recall. These evaluation metrics are defined as Equations 13–16:

(13) $$DSC=\frac{2TP}{2TP+FP+FN}$$

(14) $$IoU=\frac{TP}{TP+FP+FN}$$

(15) $$P=\frac{TP}{TP+FP}$$

(16) $$blueRecall=\frac{TP}{TP+FN}$$

where *TP*, *FP* and *FN* are defined at the pixel level, representing the counts of true positive, false positive, and false negative pixels, respectively.

### 3.3 Comparisons with other methods

To evaluate the effectiveness of the proposed method, we compared it with a variety of state-of-the-art segmentation approaches. All baseline methods are carefully re-implemented and fine-tuned on our datasets to ensure a fair comparison. The compared methods can be broadly divided into three categories:

- Traditional CNN models, including DeepLabv3 (Chen et al., 2017), DeepLabv3+ (Chen et al., 2018) and U-Net v2 (Peng et al., 2025). DeepLabv3 adopts ResNet-50 as the backbone and integrates atrous convolution to expand the receptive field without reducing spatial resolution. DeepLabv3+ extends DeepLabv3 by introducing a decoder module to recover spatial details and refine segmentation boundaries. U-Net v2 follows an encoder-decoder architecture with skip connections, emphasizing the fusion of high-level semantic features and low-level spatial details.
- Improved hybrid models, including CNN+Transformer architectures (e.g., TransUNet (Chen et al., 2024)) and CNN+Parallel Spatial Attention architectures (e.g., YOLO11n-Seg and YOLO11s-Seg (Khanam and Hussain, 2024)). TransUNet integrates Transformer’s self-attention mechanism into the U-Net framework to enhance global context modeling. The Transformer encoder captures long-range feature dependencies, while the CNN decoder preserves local spatial details. YOLO11n-Seg and YOLO11s-Seg, in contrast, adopt efficient CNN-based designs with spatial attention to strengthen feature representation and deliver both high speed and strong segmentation accuracy.
- SAM and its derived variants, including fine-tuned SAM (Kirillov et al., 2023) and SAMUS (Lin et al., 2024). SAM uses a ViT-B backbone and multi-scale feature extraction strategy. Pre-trained on 11 million images and 1 billion masks, it exhibits strong generalization ability for arbitrary object segmentation. SAMUS is an optimized SAM-based dual-branch architecture that enhances feature interaction between SAM and CNN through multi-scale feature alignment. It improves segmentation accuracy by refining boundary details and lesion integrity.

All these methods were implemented using PyTorch version 2.1.0. For DeepLabv3 and DeepLabv3+, we leveraged ResNet-50 as the backbone which is pre-trained on ImageNet. For SAM, SAMUS, and ReaLeaf-SAM, we used ViT-B as SAM’s backbone which is pre-trained on SA-1B by Meta and made the parameters of SAM trainable. For TransUNet, we adopted its hybrid CNN-Transformer architecture, specifically the R50-ViT-B 16 configuration, which is pre-trained on ImageNet. Following the models’ best settings, SAM and TransUNet’s initial learning rate is 0.0001, SAMUS’s initial learning rate is 0.0005, and all other methods’ learning rate is 0.001. All the models were trained for 300 epochs using AdamW. And these methods were trained and evaluated using 3-fold cross-validation on the same dataset.

#### 3.3.1 Model effectiveness

**Figure 6:**
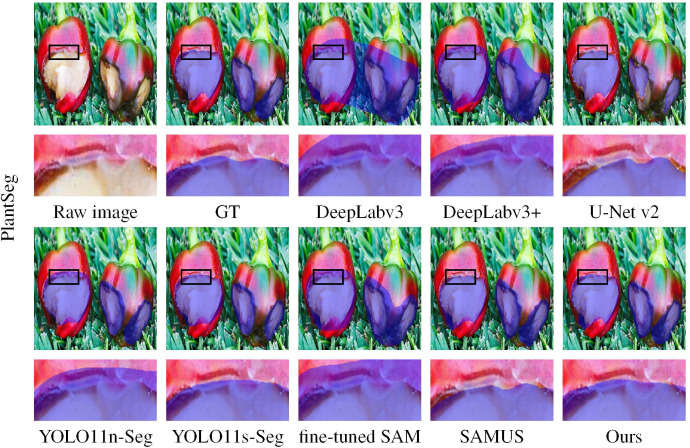
Qualitative comparison examples of a variety of state-of-the-art segmentation models on PlantSeg (bell pepper blossom end rot). Prediction masks of the lesion areas are shown in blue.

**Figure 7:**
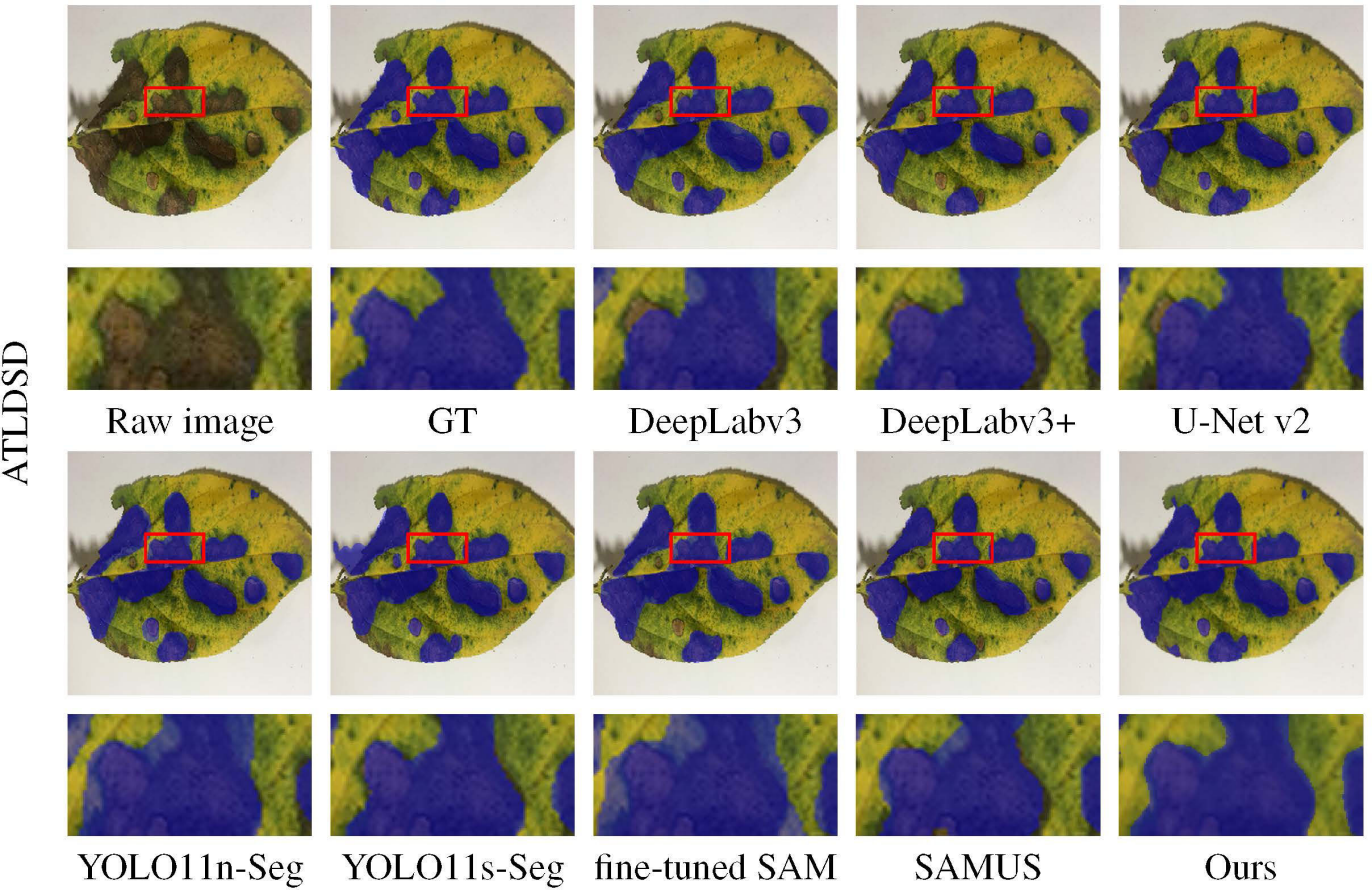
Qualitative comparison examples of a variety of state-of-the-art segmentation models on ATLDSD (apple brown spot). Prediction masks of the lesion areas are shown in blue.

**Figure 8:**
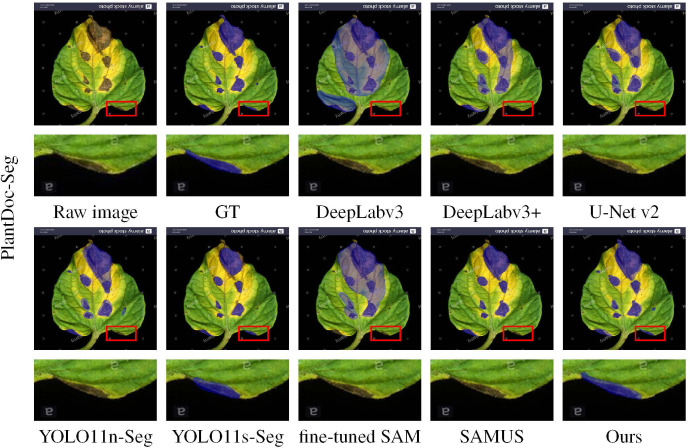
Qualitative comparison examples of a variety of state-of-the-art segmentation models on PlantDoc-Seg (tomato leaf early blight). Prediction masks of the lesion areas are shown in blue.

The quantitative results are summarized in **Table 5**, while the qualitative comparisons are shown in **Figures 6**–**8**. The proposed framework consistently achieves the best performance on all three datasets in terms of DSC, IoU, Precision, and Recall.

**Table 5:** Performance comparison between our method and other approaches.

Model	PlantSeg				ATLDSD				PlantDoc-Seg			
	DSC	IoU	P	Recall	DSC	IoU	P	Recall	DSC	IoU	P	Recall
DeepLabv3	39.02	18.66	41.59	36.34	47.94	31.70	58.18	40.27	49.71	40.05	55.25	46.36
	(± 1.23)	(± 0.97)	(± 1.58)	(± 1.52)	(± 1.04)	(± 0.87)	(± 1.44)	(± 1.21)	(± 1.34)	(± 0.92)	(± 1.27)	(± 1.58)
DeepLabv3+	44.19	29.31	46.99	40.25	54.03	38.47	61.20	46.14	58.72	47.29	63.68	52.97
	(± 1.05)	(± 0.84)	(± 1.32)	(± 1.38)	(± 0.95)	(± 0.70)	(± 1.05)	(± 1.24)	(± 1.01)	(± 0.95)	(± 1.38)	(± 1.52)
U-Net v2	50.79	42.06	53.81	46.76	63.49	56.25	70.93	55.18	66.49	54.70	75.03	55.73
	(± 0.93)	(± 0.75)	(± 1.02)	(± 1.15)	(± 0.88)	(± 0.86)	(± 0.95)	(± 1.04)	(± 0.91)	(± 0.87)	(± 1.18)	(± 1.18)
YOLO11n-Seg	67.83	65.79	72.04	62.78	80.78	73.44	85.25	73.91	80.50	77.85	83.07	76.80
	(± 0.82)	(± 0.71)	(± 0.93)	(± 0.98)	(± 0.75)	(± 0.63)	(± 0.90)	(± 0.94)	(± 0.82)	(± 0.88)	(± 0.91)	(± 1.03)
YOLO11s-Seg	77.64	66.49	80.82	73.19	85.85	80.67	87.33	82.08	87.63	82.29	89.79	83.97
	(± 0.61)	(± 0.75)	(± 0.79)	(± 0.82)	(± 0.72)	(± 0.58)	(± 0.81)	(± 0.75)	(± 0.65)	(± 0.60)	(± 0.72)	(± 0.83)
TransUNet	58.47	44.83	65.96	52.96	64.59	54.95	73.07	56.63	71.60	58.42	78.81	63.31
	(± 1.12)	(± 0.95)	(± 1.37)	(± 1.12)	(± 1.00)	(± 0.89)	(± 1.25)	(± 1.31)	(± 1.19)	(± 1.12)	(± 1.30)	(± 1.36)
fine-tuned SAM	65.53	54.15	72.69	57.42	76.08	68.95	83.74	66.56	75.59	65.04	80.97	69.37
	(± 0.90)	(± 0.73)	(± 0.95)	(± 1.13)	(± 0.91)	(± 0.82)	(± 0.93)	(± 0.98)	(± 0.89)	(± 0.80)	(± 0.89)	(± 1.05)
SAMUS	76.68	69.13	82.95	71.07	85.52	78.72	85.91	80.39	89.41	82.06	91.35	85.48
	(± 0.75)	(± 0.69)	(± 0.82)	(± 0.95)	(± 0.66)	(± 0.62)	(± 0.78)	(± 0.85)	(± 0.62)	(± 0.57)	(± 0.73)	(± 0.81)
Ours	81.05*	69.73*	85.07*	75.95*	88.27*	81.65*	88.60*	87.40*	91.12*	87.39*	97.46*	87.34*
	(± 0.57)	(± 0.42)	(± 0.64)	(± 0.69)	(± 0.51)	(± 0.46)	(± 0.61)	(± 0.75)	(± 0.58)	(± 0.49)	(± 0.58)	(± 0.65)

All results are reported as mean ± standard deviation over 3-fold cross-validation. ^∗^indicates that the improvement over the second-best method is statistically significant (*p<* 0.05, sample-wise paired t-test, *N* equals to the number of test images per dataset). Best results are in bold.

As shown in **Table 5**, traditional CNN-based models perform notably worse than our method. Specifically, DeepLabv3 shows DSC reductions of 42.03%, 40.33%, and 41.41% on the three datasets, respectively, while DeepLabv3+ and U-Net v2 also exhibit clear performance declines. This is mainly because CNNbased methods rely on local convolutions and therefore have limited ability to model long-range semantic dependencies in plant disease images.

For the hybrid models, TransUNet still underperforms our method on the three datasets, exhibiting DSC drops of 22.58%, 23.68%, and 19.52%. Similarly, YOLO11n-Seg shows drops of 13.22%, 7.49%, 10.62%, while YOLO11s-Seg records drops of 3.41%, 2.42%, and 3.49%. Although these methods enhance global feature modeling by combining CNNs with other architectures, they do not fully address the collaborative modeling of global semantics and local details, and their fusion strategies remain relatively simple.

Among SAM-based methods, fine-tuned SAM and SAMUS show DSC decreases of 15.52%, 12.19%, and 15.53%; and 4.37%, 2.75%, and 1.71%, respectively. These results suggest that fine-tuned SAM alone is insufficient for fine-grained lesion segmentation, while existing SAM-CNN frameworks still lack sufficiently effective fusion mechanisms. In contrast, our framework leverages the complementary strengths of SAM and ResNet-50 to jointly extract global and fine-grained features, while further incorporating an uncertainty encoder to estimate feature reliability and a variational fusion module to dynamically assign fusion weights. As a result, the proposed method achieves superior segmentation accuracy and robustness.

**Figure 9:**
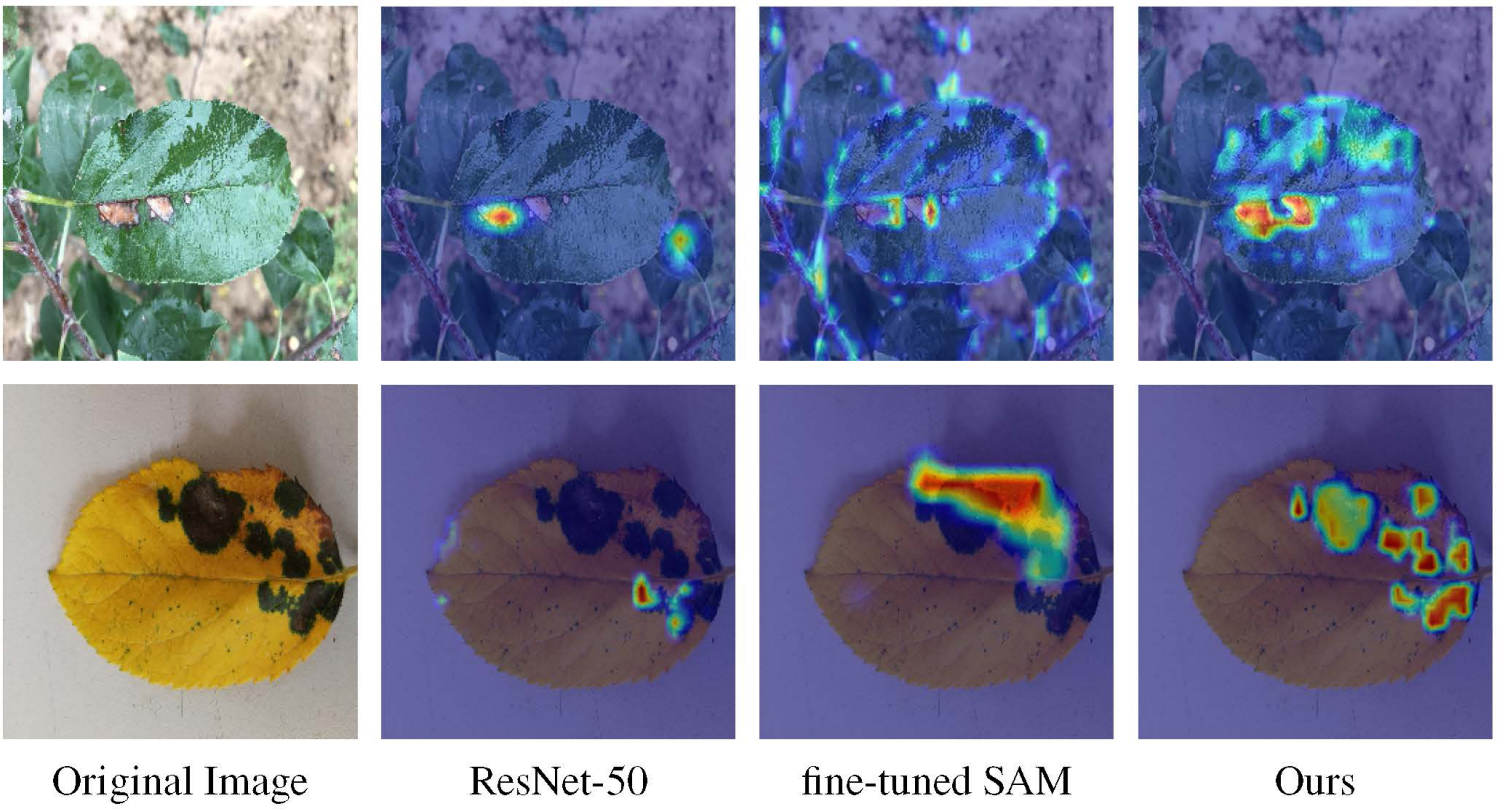
Visualization of Class Activation Maps (CAM) for ResNet-50, fine-tuned SAM, and the proposed ReLeaf-SAM.

The heatmaps shown in **Figure 9** illustrate the spatial regions attended by each model. ResNet-50 focuses on fine-grained textures but suffers from fragmented responses. Fine-tuned SAM captures global structure but lacks fine-grained details. In contrast, ReLeaf-SAM achieves the most accurate alignment with ground truth by integrating global semantics and local spatial cues.

Overall, our method achieves DSC/IoU/P/Recall scores of 81.05%/69.73%/85.07%/75.92% on PlantSeg, 88.27%/81.65%/88.60%/87.41% on ATLDSD, and 91.12%/87.39%/97.46%/87.34% on PlantDoc-Seg. These results demonstrate that the proposed framework achieves superior segmentation accuracy, boundary quality, and robustness in complex plant disease scenarios.

#### 3.3.2 Model complexity

To evaluate the model complexity, we compare ReLeaf-SAM with other models in terms of parameters, floating-point operations (FLOPs), and inference speed (FPS). The results are summarized in **Table 6**.

**Table 6:** Efficiency comparison of different methods in terms of parameters, FLOPs, and FPS, along with the DSC scores of each model on PlantSeg, ATLDSD and PLantDoc-Seg.

Model	Params (M)	FLOPs (G)	FPS	PlantSeg	ATLDSD	PlantDoc-Seg
DeepLabv3	39.6	48.3	138.3	39.02	47.94	49.71
DeepLabv3+	41.7	56.9	135.2	44.19	54.03	58.72
U-Net v2	25.1	31.8	59.4	50.79	63.49	66.49
YOLO11n-Seg	2.8	6.1	78.0	67.83	80.78	80.50
YOLO11s-Seg	10.1	15.6	84.7	77.64	85.85	87.63
TransUNet	93.2	129.5	48.4	58.47	64.59	71.60
fine-tuned SAM	90.4	103.1	42.9	65.53	76.08	75.59
SAMUS	136.2	160.1	19.8	76.68	85.52	89.41
Ours	127.5	147.6	28.3	81.05	88.27	91.12

Among all compared methods, YOLO11n-Seg is the most lightweight, with only 2.8M parameters and 6.1G FLOPs, achieving 78.0 FPS. YOLO11s-Seg (10.1M, 15.6G, 84.7 FPS) and DeepLabv3 (39.6M, 48.3G, 138.3 FPS) also exhibit high efficiency. However, their segmentation accuracy lags significantly behind our method. Fine-tuned SAM has 90.4M parameters and 103.1G FLOPs, running at 42.9 FPS. And the dual-branch SAM-CNN architecture SAMUS, requires 136.2M parameters and 160.1G FLOPs, with a lower FPS of 19.8.

ReLeaf-SAM contains 127.5M parameters and 147.6G FLOPs, running at 28.3FPS on an NVIDIA RTX 4090 GPU. Although its computational cost exceeds that of lightweight architectures such as YOLO11n-Seg (2.8M, 6.1G, 78.0FPS) and fine-tuned SAM (90.4M, 103.1G, 42.9 FPS), the gain in segmentation quality is substantial. The additional computational burden primarily stems from three key components, including a ResNet-50 detail compensation branch, an uncertainty encoder, and a variational fusion module. These designs collectively enhance the model’s sensitivity to fine-grained lesion textures and improve boundary precision in challenging field conditions. As reported in **Table 6**, ReLeaf-SAM achieves DSC scores of 81.05% on PlantSeg, 88.27% on ATLDSD, and 91.12% on PlantDoc-Seg, consistently outperforming all competing methods including fine-tuned SAM and SAMUS by a large margin. Additionally, the inference speed of 28.3 FPS is sufficient for many practical agricultural applications, including offline field diagnosis, server-based batch disease monitoring, and greenhouse scanning systems, where segmentation accuracy is the primary concern.

In summary, ReLeaf-SAM strikes an effective trade-off between accuracy and efficiency. Its representational power is stronger than that of lighter models, while its inference speed (28.3 FPS) remains adequate for real-world agricultural deployment. This makes it especially well-suited for difficult plant disease segmentation tasks characterized by cluttered backgrounds, dense leaf veins, uneven illumination, and small or early-stage lesions.

### 3.4 Ablation experiments

#### 3.4.1 Ablation experiments on different modules

To systematically evaluate the contribution of each key component, we conducted a series of ablation studies. By progressively introducing individual modules and examining the corresponding performance changes, we quantitatively assessed their effects on segmentation performance. As reported in **Table 7**, the complete model achieved the best results across all datasets.

**Table 7:** Ablation study of adding Compensation Branch (CB), Uncertainty Encoder (UE) and Variational Fusion (VF).

Module			PlantSeg				ATLDSD				PlantDoc-Seg			
CB	VF	UE	DSC	IoU	P	Recall	DSC	IoU	P	Recall	DSC	IoU	P	Recall
			64.94	53.65	72.38	57.83	75.60	68.35	83.35	71.20	76.14	65.71	81.53	69.57
✓			75.21	66.25	81.60	70.63	83.59	76.81	84.78	80.44	78.62	69.33	82.57	72.19
✓	✓		78.57	67.82	83.43	71.69	85.17	78.04	86.30	84.92	87.29	80.61	91.05	81.85
✓	✓	✓	**81.16**	**70.09**	**85.52**	**76.16**	**87.73**	**81.29**	**88.12**	**86.61**	**91.45**	**86.88**	**96.90**	**87.02**

The first row corresponds to the baseline SAM. The last row refers to the complete design of our model. Best results are in bold.

##### 3.4.1.1 Compensation branch

SAM can achieve precise localization of the entire lesion region. However, it relies more heavily on shape priors and mainly captures coarse-grained lesion contours, making it less sensitive to subtle textural differences between lesions and leaf veins. Without the compensation branch, using SAM alone achieved DSC/IoU/P/Recall scores of 64.94%/53.65%/72.38%/57.83% on PlantSeg, 75.6%/68.35%/83.35%/71.20% on ATLDSD, and 76.14%/65.71%/81.53%/69.57% on PlantDoc-Seg, as shown in the first row of **Table 7**.

To address this limitation, we introduced ResNet-50 as the compensation branch to extract highly discriminative texture features through multi-layer convolutional operations. This branch mitigates the insufficient texture perception of SAM and refines its coarse localization results. With the addition of the ResNet-50 branch, the DSC, IoU, P, and Recall improved by 10.27%, 12.60%, 9.22%, and 10.87% on PlantSeg, 7.99%, 8.46%, 1.43%, and 13.27% on ATLDSD, and 2.48%, 3.62%, 1.04%, and 3.61% on PlantDoc-Seg, respectively, as shown in the second row of **Table 7**.

##### 3.4.1.2 Variational fusion

The global features extracted by SAM and the detail features captured by the compensation branch exhibit substantial modal heterogeneity. Direct addition or concatenation can hardly exploit their complementarity and often fails to fully leverage the synergy between shape priors and texture cues. In contrast, the variational fusion module computes modal weights based on feature distribution parameters rather than conventional similarity measures, thereby adaptively assigning higher weights to more discriminative and lower-uncertainty features while suppressing responses with higher uncertainty and stronger interference.

As shown in the third row of **Table 7**, incorporating the variational fusion module into the global-detail collaborative architecture further improved model performance. On PlantSeg, DSC, IoU, P, and Recall increased by 3.36%, 1.57%, 1.83%, and 4.49%; on ATLDSD, they increased by 1.58%, 1.23%, 1.52%, and 1.63%; and on PlantDoc-Seg, they improved by 8.67%, 11.28%, 8.48%, and 8.81%, respectively. These results demonstrate that the variational fusion module can effectively alleviate the heterogeneity conflict between the global and detail features and outperform traditional linear fusion strategies.

##### 3.4.1.3 Uncertainty encoder

In real-world scenarios, such as complex backgrounds and dense leaf veins, the reliability of features extracted by the SAM and ResNet branches may fluctuate substantially. To address this issue, we introduced an uncertainty encoder, which models the global and detail features as probability distributions through explicit feature parameterization and quantitatively estimates the uncertainty of different feature responses. This provides a reliable basis for the subsequent variational fusion process to suppress high-interference and low-confidence responses while enhancing informative lesion features.

After incorporating the uncertainty encoder on the basis of the CNN branch and variational fusion, as shown in the fourth row of **Table 7**, the performance of our model was further improved across all datasets. On PlantSeg, DSC, IoU, P, and Recall increased by 2.59%, 2.27%, 2.09%, and 2.98%; on ATLDSD, they improved by 2.56%, 3.25%, 1.82%, and 3.27%; and on PlantDoc-Seg, they increased by 4.16%, 6.27%, 5.85%, and 2.74%, respectively. These results verify that the uncertainty encoder can effectively identify and suppress the interference of localized unreliable features, thereby further improving the purity and robustness of feature representations through pixel-wise uncertainty calibration.

**Figure 10:**
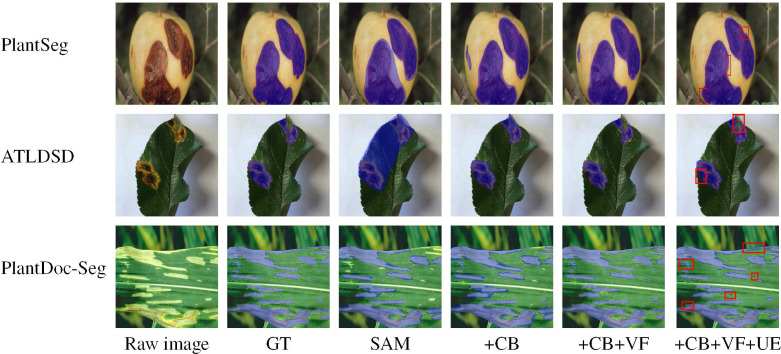
Qualitative comparison examples of adding Compensation Branch (CB), Uncertainty Encoder (UE) and Variational Fusion (VF) on PlantSeg (apple black rot), ATLDSD (apple rust) and PlantDoc-Seg (corn gray leaf spot). For each sample, we show raw image, ground truth, and results of fine-tuned SAM, SAM+CB, SAM+CB+VF, and Ours (SAM+CB+VF+UE). Prediction masks of the lesion areas are shown in blue.

The qualitative results in **Figure 10** further illustrate the progressive improvement in segmentation performance from the baseline SAM to the full model with all core modules. Although SAM can roughly localize the target regions, its predictions often suffer from missing details and coarse boundaries, resulting in limited alignment with the ground truth (GT). After introducing ResNet-50 as the compensation branch (CB), the model achieved more accurate boundary delineation and finer detail preservation, effectively alleviating SAM’s weakness in local feature modeling. Building upon this, the variational fusion (VF) module alleviated the heterogeneity between global and local features through adaptive weighting based on distribution parameters. Finally, with the addition of the uncertainty encoder (UE), the model was able to quantify feature reliability and suppress unreliable responses, leading to the best segmentation performance. As a result, the final predictions on all three datasets show high consistency with the GT, accurately preserving key structures such as lesion edges, vein structures, and leaf textures, with fewer omissions and misclassifications. These results confirm that the proposed multi-module fusion architecture effectively balances global semantics and local details, substantially improving segmentation accuracy in complex plant disease scenarios.

#### 3.4.2 Ablation experiments on different loss functions

To investigate the contribution of each loss term to segmentation performance, we conducted four groups of ablation experiments using different combinations of *L_ce_*, *L_Dice_*, and *L_KL_*, as summarized in **Table 8**.

**Table 8:** Performance comparison with different loss functions.

Loss			PlantSeg				ATLDSD				PlantDoc-Seg			
*L_ce_*	*L_Dice_*	*L_KL_*	DSC	IoU	P	Recall	DSC	IoU	P	Recall	DSC	IoU	P	Recall
✓	✓	✓	**81.16**	**70.09**	**85.52**	**76.16**	**87.73**	**81.29**	**88.12**	**86.61**	**91.45**	**86.88**	**96.90**	**87.02**
✓	✓		78.24	67.19	82.90	73.65	84.63	77.99	85.82	82.53	88.37	80.04	90.75	85.07
✓		✓	71.18	58.07	74.25	65.48	80.42	69.86	83.71	76.09	84.69	76.53	87.32	80.94
	✓	✓	76.51	65.95	82.08	70.86	83.90	74.72	85.16	81.58	87.14	78.50	89.26	84.30

Best results are in bold.

As shown in **Table 8**, the full model trained with all three loss terms, i.e., *L_ce_*, *L_Dice_*and *L_KL_*achieved the best overall performance, with DSC/IoU/P/Recall scores of 81.16%/70.09%/85.52%/76.16% on PlantSeg, 87.73%/81.29%/88.12%/86.61% on ATLDSD, and 91.45%/86.88%/96.90%/87.02% on PlantDoc-Seg. When *L_KL_*was removed, as the second row of **Table 8**, the performance dropped to 78.24% (-2.92), 67.19% (-2.90), 82.90% (-2.62) and 73.65% (-2.51) on PlantSeg, 84.63% (-3.10), 77.99% (-3.30), 85.82% (-2.30) and 82.52% (-4.08) on ATLDSD, and 88.37% (-3.08), 80.04% (-6.84), 90.75% (-6.15) and 85.07% (-1.95) on PlantDoc-Seg. The results indicate that *L_KL_*plays an important role in constraining the distribution stability of features and attention scores, thereby reducing feature unreliability caused by background interference and illumination variations, and providing a more stable basis for feature fusion.

**Figure 11:**
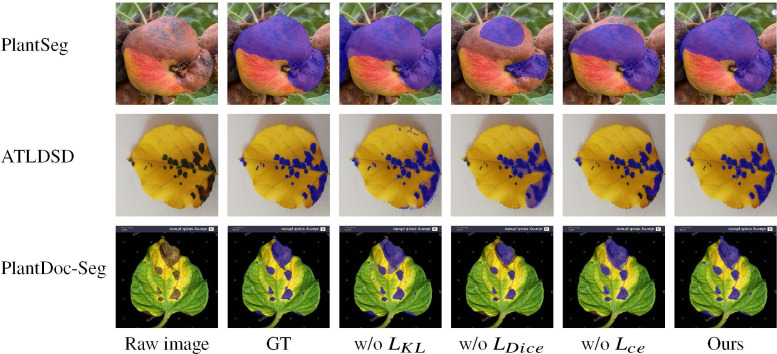
Qualitative comparison examples of different loss function groups on PlantSeg (apple black rot), ATLDSD (apple brown spot) and PlantDoc-Seg (tomato leaf early blight). For each sample, we show raw image, ground truth, and results of w/o *L_KL_*, w/o *L_Dice_*, w/o *L_ce_* and Ours (w/*L_KL_*, *L_Dice_* and *L_ce_*). Prediction masks of the lesion areas are shown in blue.

When *L_Dice_*was excluded, i.e., only *L_ce_*and *L_KL_*were retained, the performance deteriorated more severely, reaching 71.18% (-9.98), 58.07% (-12.02), 74.25% (-11.27) and 65.48% (-10.68) on PlantSeg, 80.42% (-7.31), 69.86% (-11.43), 83.71% (-4.41) and 76.09% (-10.52) on ATLDSD, and 84.69% (-6.76), 76.53% (-10.35), 87.32% (-9.58) and 80.94% (-6.08) on PlantDoc-Seg. The substantial performance degradation demonstrates the critical role of *L_Dice_*in optimizing region overlap, particularly for small lesions and sparsely distributed diseased areas. Without *L_Dice_*, the model is more prone to incomplete and fragmented segmentation, highlighting its effectiveness in alleviating class imbalance. As shown in the fourth row of **Table 8**, removing *L_ce_*reduced the performance to 76.51% (-4.65), 65.95% (-4.14), 82.08% (-3.44) and 70.86% (-5.30) on PlantSeg, 83.90% (-3.83), 74.72% (-6.57), 85.16% (-2.96) and 81.58% (-5.03) on ATLDSD, and 87.14% (-4.31), 78.50% (-8.38), 89.26% (-7.64) and 84.30% (-2.72) on PlantDocSeg. These results suggest that *L_ce_*is essential for accurate pixel-wise classification, as it helps sharpen segmentation boundaries, suppress background confusion, and improve the discrimination between lesion regions and surrounding tissues. The qualitative comparisons of different loss combinations are presented in **Figure 11**. Overall, the combination of *L_ce_*, *L_Dice_*, and *L_KL_*delivers the best segmentation performance, confirming that the three loss terms are complementary in improving region integrity, boundary accuracy, and feature stability.

#### 3.4.3 Ablation experiments with different compensation branch models

To investigate how the fine-grained feature extraction capability of the compensation branch affects the overall segmentation performance of the proposed framework, We evaluated four representative CNN architectures as the compensation branches: the U-Net encoder (referred to as U-Net) (Ronneberger et al., 2015), VGG-16 (Simonyan and Zisserman, 2014), ResNet-34, and ResNet-50 (He et al., 2016), on PlantSeg, ATLDSD, and PlantDoc-Seg. The quantitative results are presented in **Table 9**.

**Table 9:** Performance comparison with different compensation branch models, including U-Net’s CNN encoder, VGG-16, ResNet-34, and ResNet-50.

Compensation branch model	PlantSeg				ATLDSD				PlantDoc-Seg			
	DSC	IoU	P	Recall	DSC	IoU	P	Recall	DSC	IoU	P	Recall
U-Net’s Encoder	70.93	59.27	73.60	69.18	81.54	73.81	81.87	80.92	81.02	75.35	84.19	77.85
VGG-16	78.34	66.74	81.58	74.95	80.75	73.08	83.95	78.27	83.84	76.73	88.06	81.06
ResNet-34	79.48	67.91	84.65	75.53	84.20	77.84	85.76	81.06	86.37	79.11	89.59	81.99
ResNet-50	**81.16**	**70.09**	**85.52**	**76.16**	**87.73**	**81.29**	**88.12**	**86.61**	**91.45**	**86.88**	**96.90**	**87.02**

Best results are in bold.

As shown in **Table 9**, our method with ResNet-50 as the compensation branch achieved the best segmentation performance on all three datasets. Specifically, it obtained DSC/IoU/P/Recall scores of 81.16%/70.09%/85.52%/76.16% on PlantSeg, 87.73%/81.29%/88.12%/86.61% on ATLDSD, and 91.45%/86.88%/96.90%/87.02% on PlantDoc-Seg. As shown in the first row of **Table 9**, using the U-Net encoder as the compensation branch performed worst, with decreases of 10.23%, 10.82%, 11.92% and 6.98% in DSC, IoU, P and Recall on PlantSeg; 6.19%, 7.48%, 6.25% and 5.69% on ATLDSD; and 10.43%, 11.53%, 12.71% and 9.17% on PlantDoc-Seg. This is because the U-Net encoder adopts a basic convolution stacking structure without incorporating residual connections, which limits its ability to build deeper representations and capture high-level semantic features in complex scenarios. Compared with VGG-16 shown in the second row, our method with ResNet-50 improved the segmentation performance by 2.82%, 3.35%, 3.94% and 1.21% on PlantSeg, 6.98%, 8.21%, 4.17% and 8.34% on ATLDSD, and 7.61%, 10.15%, 8.84% and 5.96% on PlantDoc-Seg. Although VGG-16, with 16 layers, can extract richer mid-level features than the U-Net encoder and benefits from large-scale pre-training, it still lacks residual connections. As a result, its overall performance remains inferior to that of ResNet-34 and ResNet-50.

For the ResNet-based variants, both ResNet-34 and ResNet-50 employ residual connections. However, ResNet-34 contains only 34 trainable layers, whereas ResNet-50 increases the network depth to 50 layers through the 1 × 1-3 × 3-1 × 1 bottleneck design while effectively controlling parameter redundancy. As shown in the third and last rows of **Table 9**, ResNet-50 outperformed ResNet-34 by 1.68%, 2.18%, 0.87% and 0.63% on PlantSeg, 3.53%, 3.45%, 2.36% and 5.55% on ATLDSD, and 5.08%, 7.77%, 7.31% and 5.03% on PlantDoc-Seg. This result indicates that the deeper architecture of ResNet-50 is more effective in capturing rich high-level semantic information and fine-grained local details.

**Figure 12:**
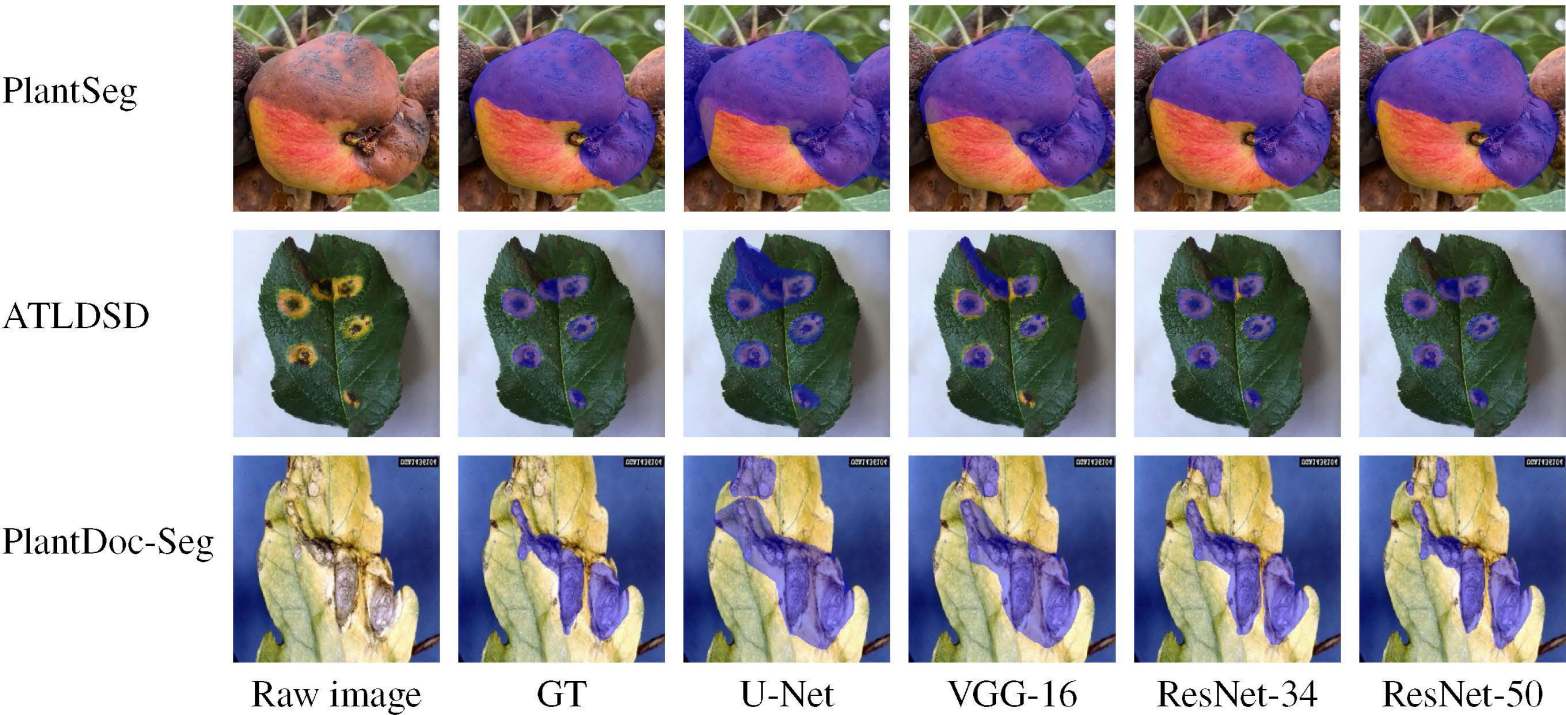
Qualitative comparison examples of different compensation branch models on PlantSeg (apple black rot), ATLDSD (apple rust) and PlantDoc-Seg (potato leaf early blight). For each sample, we show raw image, ground truth, and results of utilizing U-Net’s Encoder, VGG-16, ResNet-34, and ResNet-50 (Ours) as the compensation branch model. Prediction masks of the lesion areas are shown in blue.

The qualitative results in **Figure 12** are highly consistent with the quantitative comparisons in **Table 9**, clearly illustrating the segmentation quality differences among the four compensation branches, including the U-Net encoder, VGG-16, ResNet-34, and ResNet-50. Overall, these comparisons demonstrate that residual connections, deeper networks, and efficient feature compression are crucial for improving segmentation performance, thereby validating ResNet-50 as the most suitable compensation branch for the proposed framework.

#### 3.4.4 Ablation Experiments on Different Fusion Methods

**Figure 13:**
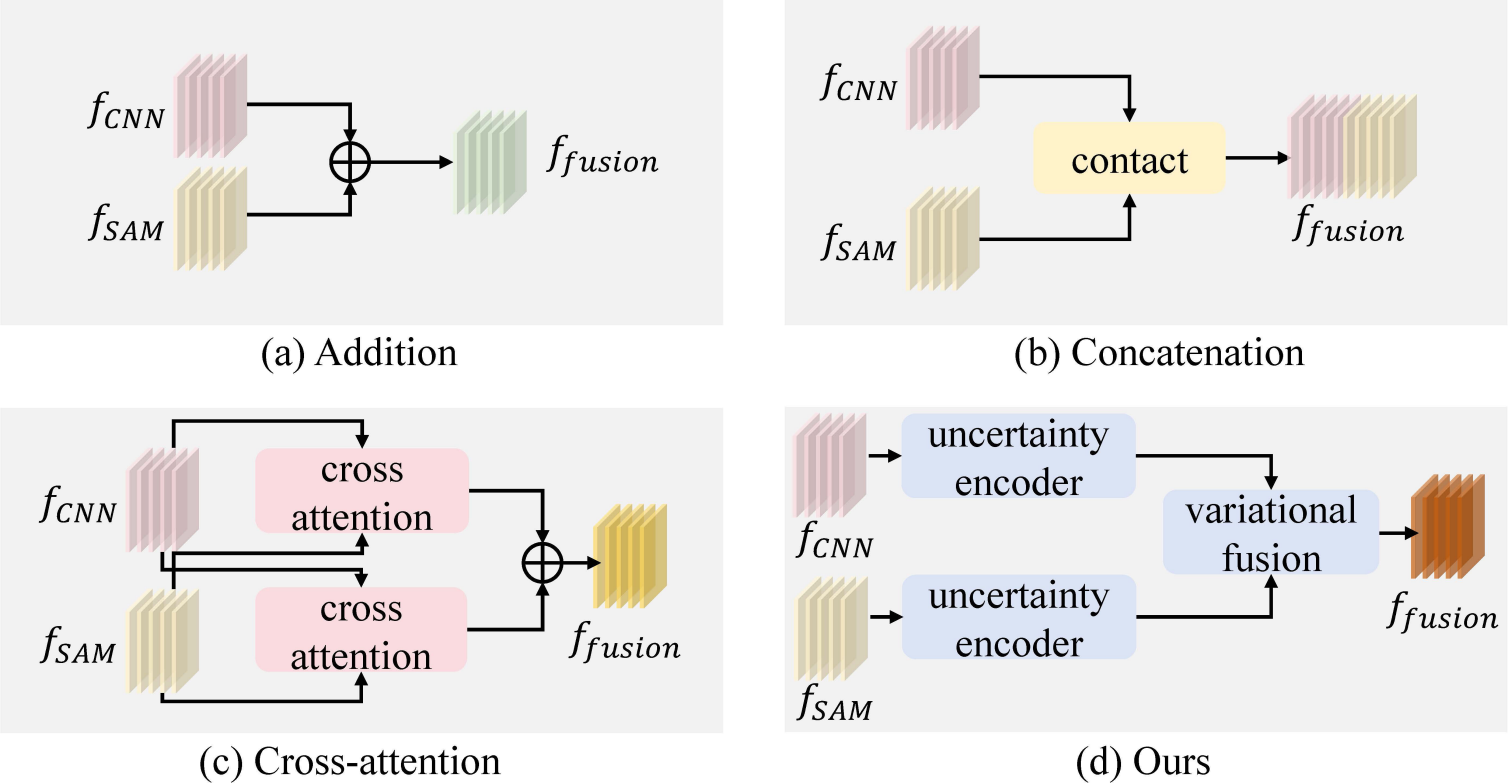
Different fusion methods between the global modeling branch and detail compensation branch, including **(a)** element-wise addition, **(b)** concatenation along the channel dimension, **(c)** cross-attention mechanism, and **(d)** the uncertainty encoder and variational attention fusion of our method.

To validate the effectiveness of the proposed fusion strategy, which incorporates an uncertainty encoder and variational attention fusion, we compared it with several representative fusion methods, including element-wise addition, concatenation, and cross-attention (Lin et al., 2022). These methods are illustrated in **Figure 13**. All variants were trained and evaluated on the same three datasets using unified metrics to ensure a fair comparison. The comparative results are presented in **Table 10**.

**Table 10:** Performance comparison with different fusion methods, including element-wise addition, concatenation, cross-attention, and our work with variational fusion and uncertainty encoder.

Fusion methods	PlantSeg				ATLDSD				PlantDoc-Seg			
	DSC	IoU	P	Recall	DSC	IoU	P	Recall	DSC	IoU	P	Recall
Addition	75.21	66.25	81.60	70.63	83.59	76.81	84.78	80.44	78.62	69.33	82.57	72.19
Concatenation	75.80	67.03	81.98	71.15	83.26	77.07	84.82	80.92	77.95	66.35	78.67	70.35
Cross-attention	77.54	68.02	82.86	71.98	84.90	77.94	85.31	81.05	83.52	78.01	86.44	76.10
Ours	**81.16**	**70.09**	**85.52**	**76.16**	**87.73**	**81.29**	**88.12**	**86.61**	**91.45**	**86.88**	**96.90**	**87.02**

Best results are in bold.

The results show that our method consistently achieves the best performance across all datasets and evaluation metrics. Specifically, it reaches DSC/IoU/P/Recall scores of 81.16%/70.09%/85.52%/76.16% on PlantSeg, 87.73%/81.29%/88.12%/86.61% on ATLDSD, and 91.45%/86.88%/96.90%/87.02% on PlantDoc-Seg. Compared with the cross-attention method, which performs second best, our approach improves DSC/IoU/P/Recall by 3.62%/2.07%/2.66%/4.18% on PlantSeg, 2.83%/3.35%/2.81%/5.56% on ATLDSD, and 7.93%/8.87%/10.46%/10.92% on PlantDoc-Seg.

This improvement can be attributed to the proposed uncertainty-aware fusion mechanism, which explicitly models feature reliability and prioritizes high-confidence information while suppressing noisy or unreliable features caused by background interference and domain shift. In contrast, cross-attention relies primarily on feature similarity, lacking an explicit reliability modeling mechanism.

Addition and concatenation show relatively limited performance. As linear fusion strategies, they only perform direct feature aggregation and fail to capture complex non-linear semantic relationships between the dual branches. Compared with element-wise addition, our method improves DSC/IoU/P/Recall by 5.95%/3.84%/3.92%/5.53% on PlantSeg, 4.14%/4.48%/3.34%/6.17% on ATLDSD, and 12.83%/17.55%/14.33%/14.83% on PlantDoc-Seg. Similarly, compared with concatenation, the improvements are 5.36%/3.06%/3.54%/5.01% on PlantSeg, 4.47%/4.22%/3.30%/5.69% on ATLDSD, and 13.50%/20.53%/18.23%/16.67% on PlantDoc-Seg. These results demonstrate that uncertainty modeling combined with variational attention provides a more effective way to fuse global modeling and detail compensation features.

**Figure 14:**
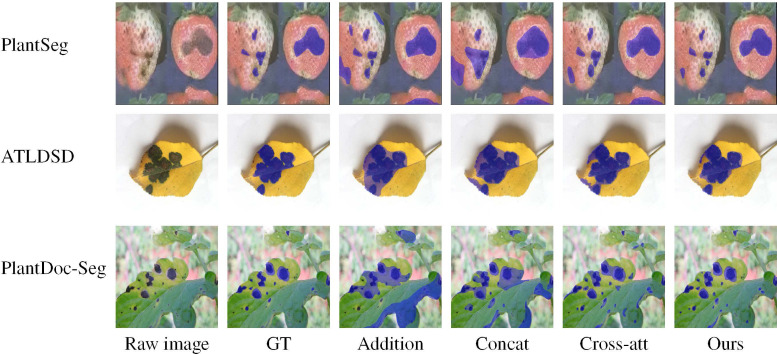
Qualitative comparison examples of different dual-branch fusion methods on PlantSeg (strawberry anthracnose), ATLDSD (apple brown spot) and PlantDoc-Seg (tomato leaf early blight). For each sample, we show raw image, ground truth, and results of utilizing addition, concatenation, cross-attention, and variational fusion (Ours) as the fusion method. Prediction masks of the lesion areas are shown in blue.

The visualization results in **Figure 14** verify the differences in segmentation performance among various fusion methods, which are highly consistent with the quantitative metrics in **Table 10**. Compared with element-wise addition, concatenation, and cross-attention fusion, the proposed method produces more complete regions and clearer boundaries. Linear fusion methods tend to lose fine details due to their limited representation capacity, while cross-attention, although stronger, still suffers from sensitivity to domain shift. By incorporating uncertainty modeling, the proposed method enables more robust integration of global (SAM) and detail (ResNet-50) features, leading to improved segmentation accuracy and stability.

### 3.5 Analysis of reliability-guided feature suppression capabilities

**Figure 15:**
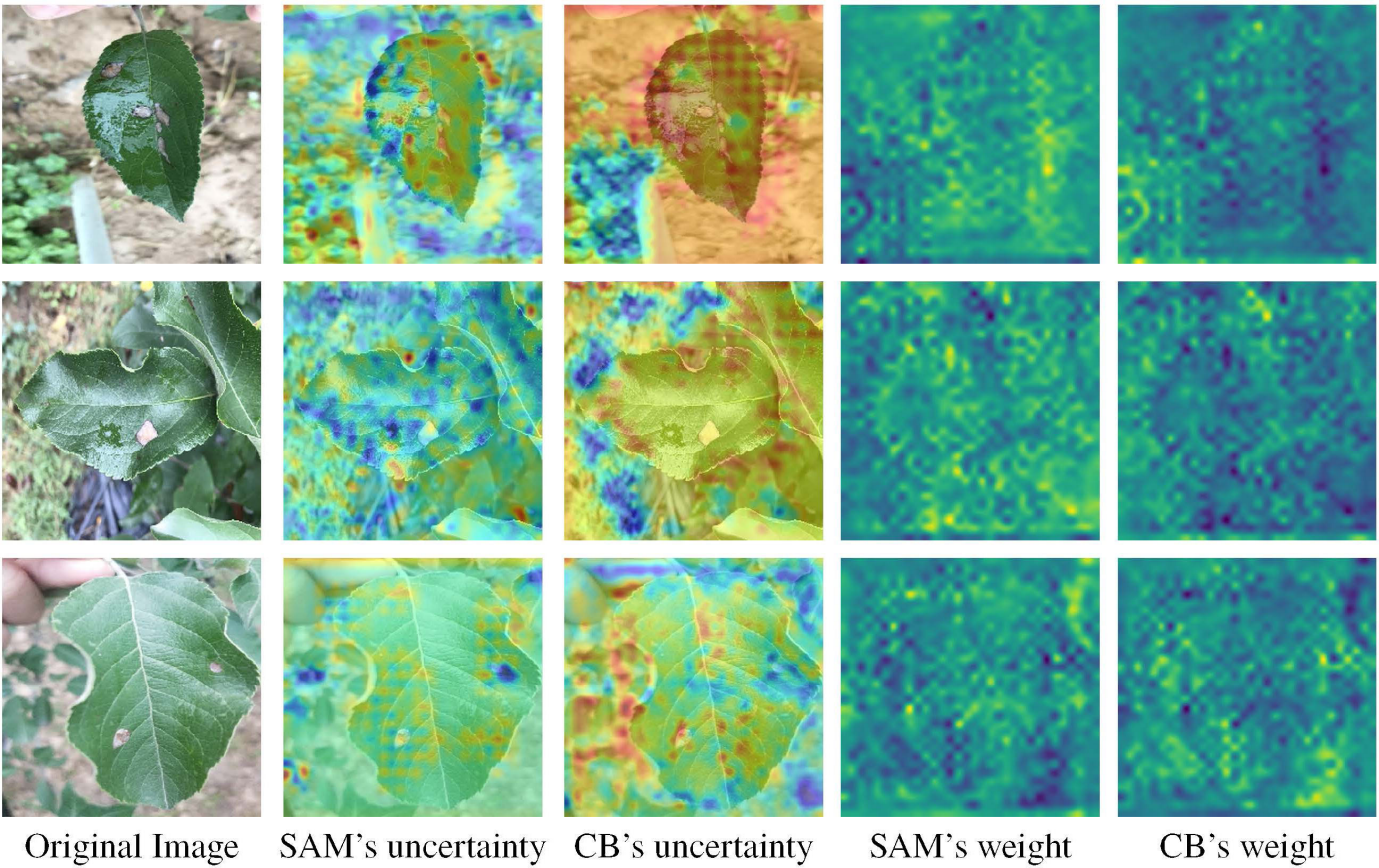
Visualization of the original image, SAM branch’s uncertainty map, compensation branch (CB)’s uncertainty map, SAM branch’s weight map, and compensation branch (CB)’s weight map. In uncertainty map, red refers to the most uncertain area, while blue refers to the most certain one. In weight map, light green refers to the highest weight, while dark blue refers to the lowest weight.

**Figure 16:**
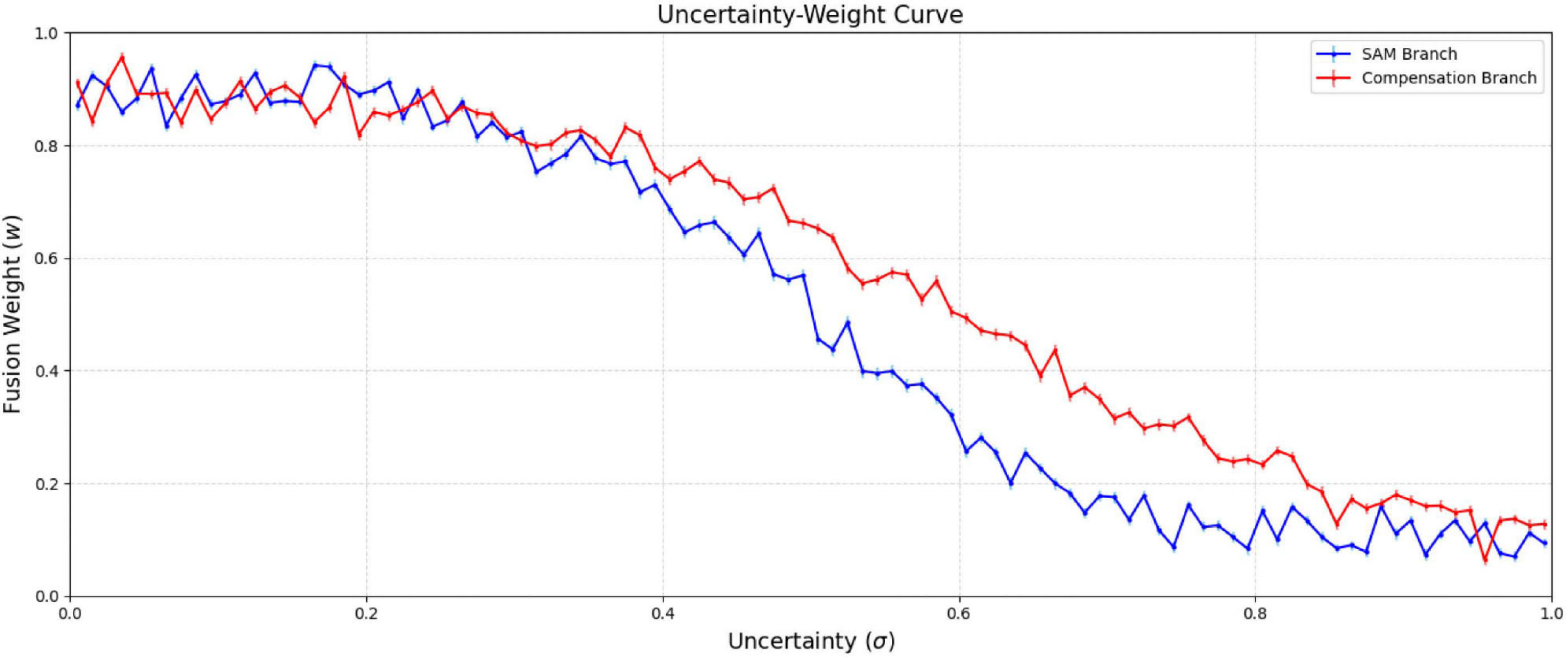
Relationship between fusion weight *w* and estimated uncertainty *σ* for the SAM and compensation branches.

To verify whether the proposed reliability-guided feature suppression capabilities, we analyze the relationship between the uncertainty output by the uncertainty encoder and the weight output by the variational fusion module. Specifically, according to Equations 2, 4, the uncertainty encoder predicts standard deviation σ*_S_* and σ*_C_* for the features extracted by the SAM branch and the compensation branch, respectively. Consequently, we use σ*_S_* and σ*_C_* to serve as pixel-level uncertainty measures for the two branches. In detail, σ*_S_*, σ*_C_*, $w_{k}^{S}$ and $w_{k}^{C}$ are upsampled by bicubic interpolation from 32×32 to 512×512 for visualization as shown in **Figure 15**. To obtain the uncertainty–weight relationship curve, we apply global linear normalization to σ*_S_* and σ*_C_*, bin the normalized values of σ*_S_* and σ*_C_*, and compute the mean weight and standard deviation within each bin. The relationship curve between uncertainty and weight is as shown in **Figure 16**.

As shown in **Figure 15**, SAM branch’s uncertainty concentrates on dense veins and ambiguous boundaries, appearing as scattered spots. It’s because SAM captures shape features but lacks fine texture discrimination, which leads to small unreliable responses in these areas. Meanwhile, the compensation branch effectively captures local details, exhibiting low uncertainty at textured regions. However, due to its limited receptive field, it struggles to model large-scale context, making it easily misled by complex backgrounds and resulting in block-wise high uncertainty. Additionally, their weight maps are consistent with the uncertainty maps, appearing dark blue in high-uncertainty regions and light green in low-uncertainty regions. Furthermore, **Figure 16** plots the relationship curve between the fusion weight and the feature uncertainty. It can be observed that the entire curve exhibits a decreasing trend. As the uncertainty increases, the corresponding weight decreases. This is consistent with our previous claims, and the result also confirms that the designed uncertainty encoder and variational fusion module successfully establish the functionality of suppressing high uncertainty and the reliability-guided capabilities of ReLeaf-SAM.

## 4 Conclusion

This paper presented ReLeaf-SAM for plant disease lesion segmentation in complex field environments. By combining SAM-based global structural modeling with ResNet-based local detail compensation, the proposed framework effectively captures both coarse lesion structures and fine-grained texture information. Furthermore, the introduced reliability-guided variational fusion mechanism enhances the collaboration between heterogeneous representations. Rather than using fixed or similarity-based fusion, our method explicitly models the confidence of dual-branch features, and adaptively emphasizes reliable lesion features and suppresses unreliable responses caused by background interference and ambiguous boundaries. Experiments on PlantSeg, PlantDoc-Seg, and ATLDSD demonstrate that ReLeaf-SAM achieves consistently strong performance across different plant species, disease categories, and imaging conditions, indicating its robustness and practical potential for real-world agricultural applications.

Despite these promising results, several limitations remain. The current framework still incurs relatively high computational and memory costs due to the joint use of SAM, ResNet-50, and probabilistic fusion modules, which drives us to focus on improving model efficiency through lightweight design and compression. In addition to model complexity, inspired by SSGAN (Zhao et al., 2025a), which effectively leverages unlabeled data to enhance performance, our future work will also explore semi-supervised learning to further reduce the dependency on expensive pixel-level annotations. Moreover, extending evaluation to more challenging field conditions and strengthening the performance of segmentation accuracy will always act as the core target of our work.

## Data Availability

The original contributions presented in the study are included in the article/supplementary material. Further inquiries can be directed to the corresponding author.

## Footnotes

1
https://www.kaggle.com/datasets/fakhrealam9537/leaf-disease-segmentation-dataset

## References

[B1] Barndorff-Nielsen O. E. (1997). Normal inverse gaussian distributions and stochastic volatility modelling. Scand. J. Stat. 24, 1–13. doi: 10.1111/1467-9469.00045 40046247

[B2] Bello R.-W. Owolawi P. A. Wyk E. Tu C. (2025). Sam-ie: Sam-enabled image enhancement for segmentation of infected cucumber leaves. Int. J. Innovative. Res. Sci. Stud. 8, 824–832. doi: 10.53894/ijirss.v8i2.5328

[B3] Bischoff V. Farias K. Menzen J. P. Pessin G. (2021). Technological support for detection and prediction of plant diseases: A systematic mapping study. Comput. Electron. Agric. 181, 105922. doi: 10.1016/j.compag.2020.105922 38826717

[B4] Chen J. Mei J. Li X. Lu Y. Yu Q. Wei Q. . (2024). Transunet: Rethinking the u-net architecture design for medical image segmentation through the lens of transformers. Med. Img. Anal. 97, 103280. doi: 10.1016/j.media.2024.103280 39096845

[B5] Chen L. Papandreou G. Schroff F. Adam H. (2017). Rethinking atrous convolution for semantic image segmentation. doi: 10.48550/arXiv.1706.05587

[B6] Chen L. Zhu Y. Papandreou G. Schroff F. Adam H. (2018). “ Encoder-decoder with atrous separable convolution for semantic image segmentation,” in Computer Vision - ECCV 2018 - 15th European Conference, Munich, Germany, September 8-14, 2018, Proceedings, Part VII, (Cham: Springer) vol. 11211 . Eds. Ferrari V. Hebert M. Sminchisescu C. Weiss Y. , 833–851. doi: 10.1007/978-3-030-01234_249

[B7] Dai W. Zhu W. Zhou G. Liu G. Xu J. Zhou H. . (2024). Aisoa-ssformer: An effective image segmentation method for rice leaf disease based on the transformer architecture. Plant Phenomics. 6, 218. doi: 10.34133/plantphenomics.0218 39105185 PMC11298559

[B8] Das A. Pathan F. Jim J. R. Kabir M. M. Mridha M. (2025). Deep learning-based classification, detection, and segmentation of tomato leaf diseases: A state-of-the-art review. Artif. Intell. Agric. 9, 100032. doi: 10.1016/j.aiia.2025.02.006 38826717

[B9] Ding X. Zhang X. Zhou Y. Han J. Ding G. Sun J. (2022). Scaling up your kernels to 31x31: revisiting large kernel design in CNNs. In: Proceedings of the IEEE/CVF Conference on Computer Vision and Pattern Recognition (CVPR 2022). (Piscataway, NJ: IEEE), 11953–11965. doi: 10.1109/CVPR52688.2022.01166

[B10] Feng J. Chao X. (2022). Apple tree leaf disease segmentation dataset. Science Data Bank. doi: 10.11922/sciencedb.01627

[B11] He K. Zhang X. Ren S. Sun J. (2016). Deep residual learning for image recognition. In: Proceedings of the IEEE Conference on Computer Vision and Pattern Recognition (CVPR 2016). (Piscataway, NJ: IEEE), 770–778. doi: 10.1109/CVPR.2016.90

[B12] Isensee F. Jaeger P. F. Kohl S. A. Petersen J. Maier-Hein K. H. (2021). nnu-net: a self-configuring method for deep learning-based biomedical image segmentation. Nat. Methods 18, 203–211. doi: 10.1038/s41592-020-01008-z 33288961

[B13] Ji S. Zhang Z. Ying S. Wang L. Zhao X. Gao Y. (2022). Kullback–leibler divergence metric learning. IEEE Trans. Cybern. 52, 2047–2058. doi: 10.1109/TCYB.2020.3008248 32721911

[B14] Khanam R. Hussain M. (2024). Yolov11: An overview of the key architectural enhancements. doi: 10.48550/arXiv.2410.17725

[B15] Kirillov A. Mintun E. Ravi N. Mao H. Rolland C. Gustafson L. (2023). Segment anything. In: Proceedings of the IEEE/CVF International Conference on Computer Vision (ICCV 2023). (Piscataway, NJ: IEEE), 3992–4003. doi: 10.1109/ICCV51070.2023.00371

[B16] Li J. Feng Q. Zhang J. Yang S. (2025). Emsam: Enhanced multi-scale segment anything model for leaf disease segmentation. Front. Plant Sci. 16, 1564079. doi: 10.3389/fpls.2025.1564079 40161224 PMC11949962

[B17] Li X. Sun X. Meng Y. Liang J. Wu F. Li J. (2020). Dice loss for data-imbalanced NLP tasks. In: Proceedings of the 58th Annual Meeting of the Association for Computational Linguistics, 465–476. doi: 10.18653/v1/2020.acl-main.45

[B18] Lin H. Cheng X. Wu X. Shen D. (2022). CAT: cross attention in vision transformer. In: International Conference on Multimedia and Expo (ICME 2022). (Piscataway, NJ: IEEE), 1–6. doi: 10.1109/ICME52920.2022.9859720

[B19] Lin X. Xiang Y. Yu L. Yan Z. (2024). Beyond adapting SAM: towards end-to-end ultrasound image segmentation via auto prompting. In: Medical Image Computing and Computer Assisted Intervention – MICCAI 2024. (Cham: Springer Nature Switzerland), 24–34. doi: 10.1007/978-3-031-72111-3_3

[B20] Liu B. Fan H. Zhang Y. Cai J. Cheng H. (2024). Deep learning architectures for diagnosing the severity of apple frog-eye leaf spot disease in complex backgrounds. Front. Plant Sci. 14, 1289497. doi: 10.3389/fpls.2023.1289497 38259944 PMC10800469

[B21] Loshchilov I. Hutter F. (2019). Decoupled weight decay regularization. In: 7th International Conference on Learning Representations (ICLR 2019). (New Orleans, LA: OpenReview.net). doi: 10.48550/arXiv.1711.05101

[B22] Lu B. Lu J. Xu X. Jin Y. (2023). Mixseg: a lightweight and accurate mix structure network for semantic segmentation of apple leaf disease in complex environments. Front. Plant Sci. 14, 1233241. doi: 10.3389/fpls.2023.1233241 37780516 PMC10535114

[B23] Lu B. Lu Y. Liang D. Yang J. (2025). Bisenext: a yam leaf and disease segmentation method based on an improved bisenetv2 in complex scenes. Front. Plant Sci. 16, 1602102. doi: 10.3389/fpls.2025.1602102 40838079 PMC12361221

[B24] Lu J. Lu B. Ma W. Sun Y. (2024). Eais-former: An efficient and accurate image segmentation method for fruit leaf diseases. Comput. Electron. Agric. 218, 108739. doi: 10.1016/j.compag.2024.108739 42321971

[B25] Madadum H. Nasir F. E. Haruehansapong K. (2025). Optimizing watermelon leaf disease detection using sam-based augmentation with yolo for practical agricultural solutions. Smart. Agric. Technol. 12, 101326. doi: 10.1016/j.atech.2025.101326 38826717

[B26] Pal A. Kumar V. Hassan K. L. Singh B. K. (2025). A framework for leaf disease analysis and estimation using maml with deeplabv3. Microsyst. Technol. 31, 715–733. doi: 10.1007/s00542-024-05686-z 30311153

[B27] Peng Y. Chen D. Z. Sonka M. (2025). U-net v2: rethinking the skip connections of u-net for medical image segmentation. In: 22nd IEEE International Symposium on Biomedical Imaging (ISBI 2025). (Piscataway, NJ: IEEE), 1–5. doi: 10.1109/ISBI60581.2025.10980742

[B28] Revathi P. Hemalatha M. (2012). Classification of cotton leaf spot diseases using image processing edge detection techniques. In: 2012 International Conference on Emerging Trends in Science, Engineering and Technology (INCOSET). (New Delhi: IEEE), 169–173. doi: 10.1109/INCOSET.2012.6513900

[B29] Ronneberger O. Fischer P. Brox T. (2015). U-net: convolutional networks for biomedical image segmentation. In: Medical Image Computing and Computer-Assisted Intervention – MICCAI 2015. (Cham: Springer), 234–241. doi: 10.1007/978-3-319-24574-4_28

[B30] Sharma N. Gupta S. Al-Yarimi F. A. M. Ghadi Y. Y. Bharany S. Rehman A. U. . (2025). Dba-deeplab: Dual-backbone attention-enhanced deeplab v3+ model for plant disease segmentation. Food. Sci. Nutr. 13, e70668. doi: 10.1002/fsn3.70668 40697703 PMC12280234

[B31] Shi D. Liang L. Chen X. Yang X. Li M. Diao M. (2026). Dtm-unet: A deep learning framework incorporating densenet and transformer for precise identification and quantitative segmentation of cucumber leaf disease. Smart. Agric. Technol. 13, 101659. doi: 10.1016/j.atech.2025.101659 38826717

[B32] Simonyan K. Zisserman A. (2014). Very deep convolutional networks for large-scale image recognition. In: 3rd International Conference on Learning Representations (ICLR 2015). (San Diego, CA, USA: OpenReview.net). doi: 10.48550/arXiv.1409.1556

[B33] Singh D. Jain N. Jain P. Kayal P. Kumawat S. Batra N. (2020). PlantDoc: a dataset for visual plant disease detection. In: Proceedings of the 7th ACM IKDD CoDS and 25th COMAD. (New York, NY: ACM), 249–253. doi: 10.1145/3371158.3371196

[B34] Singh V. Misra A. (2017). Detection of plant leaf diseases using image segmentation and soft computing techniques. Inf. Process. Agric. 4, 41–49. doi: 10.1016/j.inpa.2016.10.005 38826717

[B35] Wei T. Chen Z. Yu X. Chapman S. Melloy P. Huang Z. (2026). A large-scale in-the-wild dataset for plant disease segmentation. Sci. Data 13, 176. doi: 10.1038/s41597-025-06513-4 41663527 PMC12891534

[B36] Zhang X. Li D. Liu X. Sun T. Lin X. Ren Z. (2023). Research of segmentation recognition of small disease spots on apple leaves based on hybrid loss function and cbam. Front. Plant Sci. 14, 1175027. doi: 10.3389/fpls.2023.1175027 37346136 PMC10279884

[B37] Zhao L. Hao J. Li D. Yu J. (2025a). Semi-supervised generative adversarial network for plant leaf disease detection. Eng. Appl. Artif. Intell. 161, 112103. doi: 10.1016/j.engappai.2025.112103 38826717

[B38] Zhao L. Olivier K. Chen L. (2025b). An automated image segmentation, annotation, and training framework of plant leaves by joining the sam and the yolov8 models. Agronomy 15, 1081. doi: 10.3390/agronomy15051081 30654563

